# The Puzzling Role of Neuron-Specific PMCA Isoforms in the Aging Process

**DOI:** 10.3390/ijms20246338

**Published:** 2019-12-16

**Authors:** Tomasz Boczek, Tomasz Radzik, Bozena Ferenc, Ludmila Zylinska

**Affiliations:** 1 Department of Molecular Neurochemistry, Medical University, 92-215 Lodz, Poland; tomasz.boczek@umed.lodz.pl (T.B.); tomasz.radzik@umed.lodz.pl (T.R.); bozena.ferenc@umed.lodz.pl (B.F.); 2 Department of Ophthalmology, Stanford University School of Medicine, Palo Alto, CA 94304, USA

**Keywords:** plasma membrane Ca^2+^-ATPase, isoforms, PC12 cells, bioenergetics, Ca^2+^ signaling, aging

## Abstract

The aging process is a physiological phenomenon associated with progressive changes in metabolism, genes expression, and cellular resistance to stress. In neurons, one of the hallmarks of senescence is a disturbance of calcium homeostasis that may have far-reaching detrimental consequences on neuronal physiology and function. Among several proteins involved in calcium handling, plasma membrane Ca^2+^-ATPase (PMCA) is the most sensitive calcium detector controlling calcium homeostasis. PMCA exists in four main isoforms and PMCA2 and PMCA3 are highly expressed in the brain. The overall effects of impaired calcium extrusion due to age-dependent decline of PMCA function seem to accumulate with age, increasing the susceptibility to neurotoxic insults. To analyze the PMCA role in neuronal cells, we have developed stable transfected differentiated PC12 lines with down-regulated PMCA2 or PMCA3 isoforms to mimic age-related changes. The resting Ca^2+^ increased in both PMCA-deficient lines affecting the expression of several Ca^2+^-associated proteins, i.e., sarco/endoplasmic Ca^2+^-ATPase (SERCA), calmodulin, calcineurin, GAP43, CCR5, IP_3_Rs, and certain types of voltage-gated Ca^2+^ channels (VGCCs). Functional studies also demonstrated profound changes in intracellular pH regulation and mitochondrial metabolism. Moreover, modification of PMCAs membrane composition triggered some adaptive processes to counterbalance calcium overload, but the reduction of PMCA2 appeared to be more detrimental to the cells than PMCA3.

## 1. Introduction

The aging process is a physiological phenomenon affecting all living organisms and is associated with perpetual changes in metabolic control, the expression and stability of genes, and cellular resistance to stress. Aging itself is not a disease, but can predispose to many diseases. This process is particularly noticeable in the central nervous system (CNS), as mature neurons have no or very limited capacity for renewal. The most difficult in studies on brain aging is to separate the effects from causes, because they frequently overlap [1]. The evident hallmarks of many age-related events are calcium dyshomeostasis, mitochondrial dysfunction, oxidative stress, and impaired proteostasis [2,3,4,5]. Senescence in neurons is related to protracted elevations in cytosolic Ca^2+^ ([Ca^2+^]_c_) levels and prolonged Ca^2+^ signaling following excitatory stimulation [6,7,8]. Physiologically, intracellular Ca^2+^ ([Ca^2+^]_i_) is regulated by balancing calcium influx through voltage-gated Ca^2+^ channels (VGCC), receptor-operated calcium channels (ROCC), and store-operated calcium entry (SOCE), with active efflux by plasma membrane Ca^2+^-ATPase (PMCA), sodium-calcium exchanger (NCX), and reuptake into the endoplasmic reticulum by sarco/endoplasmic Ca^2+^-ATPase (SERCA) [9,10,11]. 

Due to high buffering capacity of mitochondria, they are also an important component for maintaining calcium homeostasis and regulation of intracellular Ca^2+^ signaling [12,13]. The key role of mitochondria is production of ATP through oxidative phosphorylation, and the major energy source in the brain is glucose. The energy produced in mitochondria is vital to meet neuronal high basal energy requirements, including maintenance of the membrane potential [14]. It is well-documented that energy metabolism decreases during aging due to overproduction of reactive oxygen (ROS) and nitrogen species (RNS), as well as deterioration of protective and repair systems [15]. In oxidative stress conditions, overproduction of ROS is expected to profoundly alter cellular metabolism and its regulation [16]. According to the free radical theory of aging, which is currently the most accepted one to explain age-associated degeneration, the products of nucleic acids, proteins, lipids, and carbohydrates oxidation are all implicated in the aging process [17]. This theory proposed by Harman [18] implicates mitochondrial membrane potential loss and concomitant decrease in ATP production as a part of a negative feedback loop toward age-associated deregulation of [Ca^2+^]_i_. 

The brain is especially vulnerable to oxidative damage but due to its high complexity, it is difficult to define precisely all the factors that trigger the aging process. It is now well established that deficits in ATP supply, which is critical for reestablishment of ion balance by the plasma membrane Ca^2+^-ATPase, can produce diverse defects in brain function. This posits disturbed calcium homeostasis in the center of several age-associated brain pathologies and should be considered as an important risk factor for the development of neurodegenerative diseases. 

## 2. Plasma Membrane Ca^2+^-ATPase in Neuronal Cells

Despite the discovery of hundreds of proteins capable of calcium buffering, one of the main roles in controlling intracellular Ca^2+^ concentration is attributed to the plasma membrane Ca^2+^-ATPase, the most sensitive cellular calcium detector. There are four main isoforms of PMCA (PMCA1–PMCA4), that are similar in structure, despite low homology at the amino acid level [19,20]. Depending on the cell and tissue type, there may be nearly 30 different PMCA variants created by alternative splicing of the original transcripts, which generates enzymes of different structural and biochemical properties, such as Ca^2+^ affinity, velocity of Ca^2+^ transport, regulatory mechanisms, or ability to interact with different signaling and regulatory proteins [21,22,23]. PMCA1 and PMCA4 are thought to be the constituent forms and are expressed in large numbers of cells [19,24]. 

The presence of PMCA2 and PMCA3 is limited almost exclusively to the excitable cells, and because of their prominent expression in the brain, they are referred to as neuro-specific [19,24,25,26]. PMCA2 and PMCA3 possess a high basal activity and belong to so-called “fast” isoforms, whereas PMCA1 and PMCA4 are much slower. PMCA, by interacting locally with a number of protein partners, can be incorporated into intracellular signaling pathways [27]. It suggests that PMCA may play a role that is far beyond its classically attributed function of a calcium transporter. In the brain, the expression profile of PMCAs changes significantly during development, confirming the unique role of each isoform [26,28,29]. Even though PMCA is primarily localized at multisynaptic endings and in a close proximity to neurotransmitter release sites (active zone) of the presynaptic terminals, particular PMCA isoforms are distributed in the brain in a region-dependent manner. It is now well documented that PMCA activity and the amount of decline with age, and concomitant elevated calcium can lead to apoptosis or necrosis, depending on the extent of metabolic stress and cytosolic Ca^2+^ overload [2,30,31,32]. Although the half-life of PMCA in the whole brain homogenate was estimated to be 12 ± 1 days, there is no available data for different PMCA isoforms and variants turnover rates [33]. Moreover, accumulation of age-dependent modifications of PMCA protein structure, despite the relatively long half-life, may alter the enzyme efficiency in Ca^2+^ extrusion [34,35,36]. 

Interesting data related to neurospecific PMCAs function were obtained after discovering that some mutations in PMCA2 are linked to hereditary deafness [37,38] and mutation of PMCA3 was found in cerebellar ataxia [39], but in each case, the total efficiency of Ca^2+^ extrusion was reduced. It may suggest that impaired PMCA2 or PMCA3 function cannot be simply substituted for other isoforms. Using several models, including the adult and aged mouse brain, Alzheimer’s disease-affected human brain, SH-SY5Y human neuroblastoma cells, as well as purified synaptosomal PMCA from pig cerebrum, Mata’s group discovered that PMCA activity was inhibited by tau protein and amyloid-β peptides, and PMCA4b appeared to be the most sensitive target for that inhibition [40,41,42,43]. Moreover, CaM exhibited a protective action against functional impairment of PMCA by tau and Aβ peptide. Thus, although PMCA2 was shown to be more resistant to amyloid-β inhibition, its downregulation in the aging brain, together with lowered PMCA4 activity and the CaM amount, could be responsible for progressive development of AD symptoms.

One of the widely used in vitro model of sympathetic neurons are differentiated PC12 cells. This line was isolated from the pheochromocytoma tumor of rat adrenal glands that have an embryonic origin from the neural crest, which gives PC12 cells unique ability to acquire a number of features characteristic for mature neurons [44]. Upon treatment with nerve growth factor (NGF), they cease proliferation, start to extend neurite-like protrusions, express functional neuronal membrane channel, develop action potentials, and respond to acetylcholine treatment. Similar effects can be triggered by dibutyryl cyclic adenosine-3′, 5′-monophosphate (db-cAMP) [45,46]. Whereas in undifferentiated PC12 cells, all main PMCA isoforms have been detected, with PMCA4b constituting a major one, the differentiation process stimulates the expression of additional splice variants (i.e. 1c, 2a, 2c, 4a) although with unknown physiological function [47,48]. 

To evaluate the possible contribution of neuro-specific PMCA isoforms to the aging process, we developed PC12 lines deficient in PMCA2 (­PC12_2) or PMCA3 (PC12_3). Our extensive functional and molecular analyses gave new insights into downstream cellular processes that can be modified by altered PMCA composition. 

## 3. Differentiated PC12 Cells as a Model of Aging Neuron

The knockdown of PMCA isoforms was done using eukaryotic vectors containing antisense sequences designed to either PMCA2 or PMCA3, and in stably transfected PC12 lines the protein level of each isoform was reduced by almost 50% [49]. Staining of PMCA-deficient cells with the marker protein of growth cones showed the absence of a central structure surrounded by F-actin-rich filopodia and lamellipodia and strong condensation of this protein in neuronal terminals, suggesting that growth cones can be in the retraction phase. Reduced growing potency and neurite retraction are characteristic for aged neurons and frequently observed in response to injury or disease [50,51].

A direct functional consequence of a partial loss of each isoform was a moderate, but sustained, increase in resting Ca^2+^ concentration by 30–40 nM. In addition, PMCA2 deficiency increased the percentage of cells in the late apoptotic phase, which was detected by AnnexinV/propidium iodide assay and confirmed by the observed DNA fragmentation. Similar effects were reported for rat primary neurons and human SH-SY5Y neuroblastoma cells transfected with PMCA2 siRNA [52]. Beside higher basal Ca^2+^ level and impaired Ca^2+^ clearance following stimulation, these authors also demonstrated more intensive cell death upon exposure to excitotoxic concentrations of agents rising intracellular Ca^2+^. The correlation between PMCA2 down-regulation and disturbances of cell function, including the augmented cell death, was reported in neurons, which strongly implicated a protective PMCA2 role [53,54,55,56,57]. 

Although reduction of “fast” PMCA isoforms affected functionality of the cells, it did not change the total amount of PMCA protein which could be due to up-regulation of PMCA1 isoform in both PMCA-deficient lines, and PMCA4 that increased only in response to PMCA3 silencing [49]. Nevertheless, elevated cytoplasmic Ca^2+^ concentration indicated that these adaptive changes were insufficient to fully substitute even for the partial loss of PMCA2 or PMCA3 function. However, in the PC12_3 line, compensatory up-regulation of PMCA4 seemed to counteract Ca^2+^-dependent induction of apoptosis more effectively, suggesting that PMCA3 deficit could be less detrimental for intracellular calcium homeostasis. 

Another protective mechanism aimed against Ca^2+^ cytotoxic effects was up-regulation of SERCA2 and SERCA3 isoforms in both PMCA-deficient lines [49]. It correlated with higher accumulation of Ca^2+^ in the endoplasmic reticulum. SERCA family includes three members (SERCA1–3), and two splice variants of SERCA2. SERCA2b is ubiquitously expressed, while SERCA2a and SERCA3 exhibit more discrete localization [58,59]. Since SERCA’s half-life (10–14 days) has been suggested to be similar to PMCA [2], and energetic cost of calcium transport by SERCA is lower than that of PMCA (2Ca^2+^/ATP *vs*. 1Ca^2+^/ATP, respectively) [60], this compensatory effect observed in PC12 cell lines could provide long-term multifaceted protection against calcium cytotoxicity. However, the molecular mechanism(s) of the interplay between these calcium pumps remains a mystery.

Further analysis of the effects of reduced PMCA2 or PMCA3 expression on the kinetics of Ca^2+^ extrusion showed a significantly increased influx and longer half time (t_1/2_) of Ca^2+^ clearance after KCl-induced depolarization [49]. In excitable cells, membrane potential-dependent [Ca^2+^]c raises are generated mainly by the voltage-gated Ca^2+^ channels (VGCCs), of which four types: N, L, P/Q, and T are present in PC12 cells [61,62]. The profiling of VGCC expression showed elevated transcript level of P/Q and L types in both PMCA-deficient lines and the T-type solely in PMCA2-deficient cells. Functional analysis with specific pharmacological inhibitors revealed that higher expression of these VGCCs corresponded to their increased contribution to the total Ca^2+^ load induced by KCl. 

It has been widely documented that during aging, ion channel dysfunctions participate in the generation of ionic dyshomeostasis, alteration of membrane potential as well as signal transduction pathways, which can eventually modify cellular physiology [63]. Earlier studies have showed that the aging-related increase in Ca^2+^-mediated responses depended on greater activity of L-VGCC [64,65]. Moreover, a growing body of evidence indicated an increase in the expression and function of L-type Ca^2+^ channel with aging [66,67,68,69], and higher L-VGCC density in the hippocampus was positively correlated with cognitive impairment in aged animals [62]. An interesting observation was an up-regulation of T-type VGCC in response to PMCA2 depletion and its increased contribution to Ca^2+^ influx. Physiologically, T-type channels regulate neuronal excitability, differentiation, growth, and proliferation [70]. An important link was shown between T-type calcium channels and long-term potentiation (LTP) [71]. Downregulation of the T-type VGCC was demonstrated in the mouse model of Alzheimer’s disease [72]. It was also reported that a closing time in some populations of these channels is significantly longer than the other VGCC [72]. Therefore, one can hypothesise that together with a reduced Ca^2+^ extrusion, elevated presence of T-type channels in PMCA2-deficient line might contribute to sustained increase in [Ca^2+^]_i_. 

Based on this data, it can be assumed that higher amplitude of [Ca^2+^]_c_ rises during depolarization reflects the presence of more functional calcium channels in the plasma membrane. Activation of individual channels and coupling their functions with downstream cellular signaling pathways, can induce a different response e.g., by modulation of specific genes expression. In addition, changed VGCC profile in response to manipulations in PMCA expression strongly suggests a transcriptional link between these two opposing systems of Ca^2+^ transport.

The changes in main components of calcium handling systems described above revealed some differences in the cell response, which seemed to be driven by the presence of particular PMCA isoform (Table 1). Interestingly, knockdown of PMCA2 reproduced a wider spectrum of morphological and molecular abnormalities observed during physiological brain aging. 

## 4. The Interplay between PMCA, Calcium, and Mitochondrial Function during Aging

Mitochondria are central hubs in neuronal pathology integrating energy production, Ca^2+^ homeostasis, cell signaling, and controlled cell death [73]. Preservation of these functions is especially important in the brain as neurons contain a large number of mitochondria to fulfill energy needs for synaptic processes. The decrease in mitochondrial functionality in the brain, especially in the hippocampus, is associated with the normal aging process and progressive loss of synaptic function [74]. Changes in mitochondrial activity during aging have also been associated with increased mitochondrial ROS production, causing cellular damage of mitochondrial and nuclear DNA and advancing age-related diseases [75]. 

One of the most important regulators of mitochondrial function in aging is the second messenger Ca^2+^. Buffering of [Ca^2+^]_i_ by mitochondria increases when cells age, as was demonstrated in peripheral sympathetic neurons and intestinal smooth muscle cells [76,77]. The regulation of [Ca^2+^]_I_ by mitochondria seems to be especially relevant in neuronal cells, in which local Ca^2+^ uptake by neighboring mitochondria regulates the duration of cytosolic Ca^2+^ fluxes generated by the influx through plasma membrane calcium channels [78]. The possibility that changes in intracellular Ca^2+^ homeostasis might explain mitochondrial impairments in senescent cells arise from calcium hypothesis of neuronal aging [79]. According to this hypothesis, a change in any of the Ca^2+^ transporting systems over a long time period should affect [Ca^2+^]_i_. To confirm it, several research groups showed elevated resting [Ca^2+^]_i_ and reductions in the amplitude of stimulation-induced [Ca^2+^]_i_ signal in aged neurons [80,81]. A similar effect on Ca^2+^ homeostasis was observed by us in response to PMCA2 or PMCA3 knockdown, pointing out a slower calcium clearance following Ca^2+^ loads, which is a common hallmark of aging cells. In PMCA-deficient cells, higher resting [Ca^2+^]_i_ may directly reflect a partial loss of calcium clearing potency and sensitize mitochondria to buffer cytosolic calcium transients even in a condition of moderate increases in [Ca^2+^]_i_. 

It is expected that accumulation of Ca^2+^ by mitochondria will stimulate Ca^2+^-dependent dehydrogenases of the TCA cycle, providing reducing equivalents to boost the activity of respiratory chain and thereby, ATP production via oxidative phosphorylation (OXPHOS) [82]. The ATP supply is then needed for restoration of ion gradient across the plasma membrane by PMCA or Ca^2+^ transport to the ER by the SERCA pump. However, in the case of mitochondrial overwhelming with Ca^2+^, permeability of the inner mitochondrial membrane increases dramatically, resulting in the dissipation of mitochondrial membrane potential, loss of mitochondrial respiration, and finally, initiation of cell death [83,84]. Large dissipation of mitochondrial gradient was observed in PMCA2-deficient cells during massive Ca^2+^ entry, an event that was blocked by cyclosporine A or bongkrekic acid, suggesting the involvement of mitochondrial permeability transition pore (mPTP). The mPTP is a nonselective channel located in the inner mitochondrial membrane that allows the release of excessive Ca^2+^ accumulated in mitochondria. However, overload of the mitochondrial matrix with Ca^2+^ or ROS may trigger mPTP prolonged opening and liberation of small molecules and pro-apoptotic factors, finally causing membrane potential loss, reduction in ATP level, and eventually, cell death [85,86]. Similar to the sequence of events demonstrated in PMCA2-deficient cells during depolarization, Scorrano and coworkers showed that in vitro depolarization induced mPTP opening when mitochondria were suitably loaded with Ca^2+^ [87]. The explanation of this phenomenon is likely to involve the enhancement of ROS formation, what can be suggested in these cells by a higher level of GSH as an adaptive response to free radicals overload [88]. 

An enhanced susceptibility to mPTP opening and the loss in PMCA activity in synaptic membrane are both reported in the aged brain [89]. In contrast to the catastrophic nature of the mPTP opening, the mitochondrial membrane was able to repolarize in PMCA2-deficient cells upon stimulus withdrawal, pointing out the ability of the electron transport chain (ETC) to rebuild the proton gradient and restore membrane potential. Because even partial loss of PMCA2 led to higher percentage of apoptotic cells [49], it is plausible that only partial or brief opening of mPTP was sufficient to drive cell death. Recent comparative studies showed a decreased threshold for Ca^2+^ concentration that induced mPTP opening in mitochondria isolated from old rats [90]. Therefore, it is possible that PMCA2 loss and concomitant changes in the cytolic/mitochondrial calcium circuit can increase the probability of mPTP opening at lower rises in [Ca^2+^]_i_. Whether mPTP is involved in the physiological aging process is still under debate, but the latest data demonstrated reduced mitochondrial calcium buffering capacity and increased sensitivity to mPTP formation in putamen of aged monkeys [91]. 

Neurons rely almost exclusively on the mitochondrial OXPHOS system to generate ATP and the primary role of mitochondrial calcium ([Ca^2+^]_m_) is to boost energy production. During respiration, electrons from reducing equivalents (NADH and FADH_2_) are transferred through ETC complexes (complex I-V) and H^+^ are pumped out to intermembrane space, creating an electrical (ΔΨm) and pH (ΔpH) gradients, both thermodynamically equivalent to drive ATP synthesis. Formation of ΔΨm is fundamental for Ca^2+^ uptake to mitochondria and adequate mitochondrial function, mainly for ATP synthesis by the F_0_F_1_ATP synthase [92]. Mitochondria also buffer cytosolic Ca^2+^ elevations caused by the influx through plasma membrane Ca^2+^ channels [93] or by depletion of the internal Ca^2+^ stores [94]. The ER together with the mitochondria located in regions so called mitochondria-associated ER membranes (MAMs) form highly sophisticated toolkits enabling Ca^2+^ trafficking between these organelles [95]. 

It has been demonstrated that PMCA2 and PMCA4 are tethered to specialized calcium microdomains located at the plasma membrane through the interaction with postsynaptic density protein 95 (PSD-95) [96]. PMCA/PSD-95 complexes further recruit N-methyl-D-aspartate (NMDA) receptor subunits R1 and R2A to form multiprotein aggregates [97]. By bringing PMCA to a close proximity of Ca^2+^ entry sites, formation of such structures can allow for rapid and effective response to local Ca^2+^ increases. Disrupted NMDA/PSD-95 interaction, observed in animal models of several neurological diseases [98,99], is expected to result in receptor hypersensitivity and excitotoxicity, ultimately leading to cell death. Many important studies suggest that Ca^2+^ uptake by the mitochondrial uniporter (mtCU) plays an essential role in NMDA receptor activity-driven excitotoxicity [100,101,102]. NMDA receptor stimulation was found to increase mitochondrial membrane depolarization in neurons overexpressing mtCU, whereas knockdown of endogenous mtCU reduced NMDA-induced increases in mitochondrial Ca^2+^, making cells resistant to excitotoxicity [103]. In the scenario of NMDA receptor-induced and Ca^2+^-mediated mitochondrial swelling referred to as “mitochondrial aging”, progressive loss of respiratory control is in fact due to abrupt increase in mPTP conductance. Increased permeability of mitochondrial membrane initiated at the level of individual mitochondria is spread across increasing fraction of the mitochondrial population (for comprehensive review see [104]), and usually leads to a massive cell death. Such mode of neuronal death triggered by overstimulation of NMDA receptors is associated with cerebral ischemia and neurodegenerative diseases [105,106,107].

The mtCU complex consists of pore-forming subunit (MCU) and several regulatory subunits (MICU1, MICU2, MICU3, MCUb, and EMRE) [108,109,110]. Mitochondrial calcium uptake 1 and 2 (MICU1 and MICU2) form a central semiautonomous assembly [111] to establish a threshold for MCU opening, keeping it closed at nanomolar [Ca^2+^]_c_, thus protecting mitochondria from Ca^2+^ overload [112]. Elevations of [Ca^2+^]_c_ above 2–3 µM are expected to trigger channel opening and promote cooperative increase in Ca^2+^ uptake as described in earlier studies [113,114]. The recent studies on MICU1^−/−^ mouse showed that in purified mitochondria, the MICU1 absence augmented calcium uptake at low [Ca^2+^]_c_ but inhibited the uptake rate when [Ca^2+^]_c_ increased [115]. Young MICU1^−/−^ mice had underdeveloped cerebellum, abnormal persistence of the outer granular layer at postnatal day 12, and altered arborization of Purkinje cells. Interestingly, surviving MICU1^−/−^ mice appeared to improve over time and previous histological abnormalities seen in cerebellum were resolved [115]. In addition, the differences in resting calcium, ATP level, and muscle lactate were no longer visible, although MICU1^−/−^ mice did continue to have neurological and muscular defects. The authors suggested that such gradual improvement is due to age-related functional remodeling of mitochondria, which has been recently demonstrated in mouse with reduced MCU activity [116]. In the case of MICU1 deletion, this remodeling appears to involve alterations in the expression ratio of MCU to EMRE.

EMRE is important for Ca^2+^ uptake and bridges the calcium-sensing role of MICU1 and MICU2 with the calcium-conducting function of MCU [109]. It seems to act as scaffold for the correct stoichiometric assembly of the complex and in its absence, uniporter current is lost despite proper MCU expression and oligomerization. Liu and colleagues [115] observed reduced EMRE expression in older MICU1^−/−^ mice, which correlated with phenotypic improvement. They were, however, unable to obtain MICU1^−/−^ EMRE^−/−^ mice due to compromised survival. Nonetheless, deletion of one allele of EMRE on the background of MICU1^−/−^ resulted in reduced calcium uptake at both low and high extra-mitochondrial calcium levels. Consistent with that, matrix Ca^2+^ level in brain mitochondria was similar between wild type and MICU1^−/−^ EMRE^+/−^ mice. This genetic background also produced age-appropriate cerebellar morphology but when compared to MICU1^−/−^, MICU1^−/−^ EMRE^+/−^ mice exhibited phenotypic and behavioral improvement. In neurons, non-assembled EMRE is degraded by m-AAA protease, which ensures sufficient assembly of gatekeeper subunits with MCU [117]. Very recently, it has been demonstrated that accumulation of constitutively active MCU-EMRE channels lacking gatekeeper subunits due to loss in m-AAA protease led to mitochondrial Ca^2+^ overload, mPTP opening, and subsequent neuronal death [117].

The function of other mitochondrial uniporter subunits is currently under intensive investigation. When looking at the tissue-specific MCU complex composition, MICU3 is predominantly expressed in the central nervous systems, although its low levels were also detected in skeletal muscle [118,119]. It can form heterodimers with MICU1 but not MICU2, but the function is opposite to MICU2, as its expression promotes mitochondrial Ca^2+^ accumulation at high [Ca^2+^]_c_. Therefore, MICU3 is considered to act as a highly potent cooperative MCU activator with no gatekeeping function. Patron and colleagues [120] have recently performed the extended analysis of MICU3 function in neurons. They found that MICU1/MICU2 dimers ensured low Ca^2+^ cycling in the steady-state conditions whereas MICU1/MICU3 dimers activated MCU-dependent Ca^2+^ uptake even in the low [Ca^2+^]_c_. MICU3, when silenced, was also the only MICU isoform that effectively decreased mitochondrial Ca^2+^ transients evoked by synaptic activity [120]. 

In principle, aged neurons undergo considerable changes in Ca^2+^ store content, mtCU expression, and ER-mitochondria domains [121]. Increased mtCU over time is thought to respond to higher Ca^2+^ release from the ER, which is in line with the observation of increased mitochondrial-ER coupling in aging in the face of mitochondrial depolarization [121]. In view of that, silencing of MICU3 could potentially slow down age-associated mitochondrial deficits by decreasing the amplitude of [Ca^2+^]_c_ rises, as shown by Patron et al. [120], while MICU2 knockdown is expected to exert the opposite effect.

**Figure 1:**
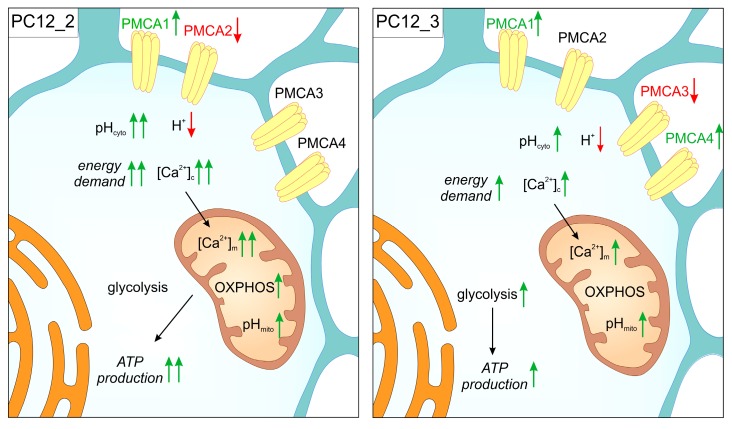
Differences in mitochondrial function in PMCA-deficient PC12 cells. Higher resting [Ca^2+^]_i_ that reflected a partial loss of calcium clearing potency might sensitize mitochondria to buffer cytosolic calcium transients. Because PMCA operates as a 1Ca^2+^/1H^+^ antiporter, the extrusion of Ca^2+^ introduces large quantities of H^+^ that are readily transported to mitochondria. Due to alkalization of mitochondrial matrix in steady-state conditions and because pH_cyto_ was higher, but still lower than pH_mito_, in the cells increased pH gradient which could drive a higher rate of ATP synthesis, more significantly in PC12_2 cells. The loss of fast acting PMCA2 could stimulate other ATP-dependent Ca^2+^ extruding proteins to restore [Ca^2+^]_c_, thereby increasing cellular demand for ATP. Also, in this line, OXPHOS rather than anaerobic glycolysis was a main energy supplying pathway. Interestingly, knockdown of PMCA3 isoform did not change the rate of OXPHOS. However, enhanced lactate release and greater sensitivity of the ATP level to glucose withdrawal in the presence of pyruvate suggests the reliance on anaerobic glycolysis, so the local synthesis of ATP may be sufficient to provide an adequate amount of energy. Other details are in the text. PMCA - plasma membrane Ca^2+^-ATPase.

Overall, the effect of higher [Ca^2+^]_m_ is expected to coordinate upregulation of OXPHOS machinery resulting in faster respiration and ATP production (Figure 1).

The recent report [122] showed that the basal rate of mitochondrial respiration was increased in aged endothelial cells, indicating enhanced mitochondrial contribution to energy balance in steady-state conditions and age-related increase in ER-mitochondrial Ca^2+^ transfer. In our study, a positive correlation between oxygen consumption and the ATP level was observed in PMCA2-deficient cells which indicates higher energy demands. Indeed, the experiments with KCN and oligomycin showed that OXPHOS rather than anaerobic glycolysis is a main energy suppling pathway in these cells. This was further supported by higher basal glucose consumption and lower NAD(P)H level accompanied by increased activity of proton-pumping ETC complexes (I, III, and IV). These findings are in agreement with the boosting effect of Ca^2+^ on mitochondrial metabolism and suggest that the rate of OXPHOS in PMCA2-deficient cells may be controlled at the activity of ETC proteins. Interestingly, knockdown of PMCA3 isoform did not change the rate of OXPHOS. However, enhanced lactate release and greater sensitivity of the ATP level to glucose withdrawal in the presence of pyruvate suggests the reliance on anaerobic glycolysis. This brings up an intriguing question about preferential fueling of PMCA isoforms by different energy-generating pathways. It is tempting to hypothesize that the loss of fast acting PMCA2 will stimulate other ATP-dependent Ca^2+^ extruding proteins to restore [Ca^2+^]_i_, thereby increasing cellular demand for ATP. When PMCA3 was depleted, [Ca^2+^]_c_ rise was only moderate, so the local synthesis of ATP by glycolysis may be sufficient to provide adequate amount of energy (Figure 1). 

Whatever the explanation might be, increased [Ca^2+^]_i_ as a result of PMCA2 or PMCA3 knockdown seems to be central for understanding the specific coupling between PMCA2 and mitochondrial metabolism of aging neurons. Our results of increased mitochondrial activity, coexisting with higher Ca^2+^ accumulation in the ER, as described in a previous chapter, are consistent with the recent hypothesis of enhanced ER-mitochondrial Ca^2+^ influx during aging and are further supported by in vitro studies based on rat hippocampal neurons [121]. Although the exact mechanism is not known, the mild ER stress due to increase in ER loading with Ca^2+^ observed in PMCA-deficient cells, which yet does not induce massive cell death, can eventually cause slow Ca^2+^ leak and activation of proteins involved in ER-mitochondrial tethering. This crosstalk might help to sustain elevated mitochondrial ATP production, creating a positive feedback loop temporarily prolonging ER function during aging. Based on the biphasic model of mitochondrial aging reported for primates and rodents, increased respiration and activity of the ETC complexes in PMCA2-deficient cells may reflect the early stage in which Ca^2+^-dependent increased mitochondrial activity boosts the generation of harmful ROS. As the aging progresses, ROS accumulation combined with a loss of scavenging mechanisms negatively affect the activity of ETC, leading to severe mitochondrial dysfunction [123], which has been widely reported as a hallmark of ageing. Strong experimental data seem to support this hypothesis, as increased expression of mitochondrial genes of complexes I, III, IV, and V was reported in 18-month old mice in the hippocampus, medial prefrontal cortex, and striatum [124], whereas later in aging, the expression of ETC proteins as well as the activity of complexes I and IV decreased in substantia nigra, hippocampal dentate gyrus, frontal cortex, and cerebellum [125,126].

The fundamental question in respect to higher mitochondrial activity in PMCA2-deficient cells is how the driving force for ATP synthesis is generated. The findings of Poburko et al. [127] showed that bursts of proton accumulation in the cytosol generated by the activity of PMCA were readily transmitted to mitochondria. A similar relationship between pH_mito_ and pH_cyto_ was previously demonstrated in MDCK cells and was attributed to the activity of mitochondrial proton antiporters [128]. The pH_mito_ recordings suggested that rapid equilibration of mitochondrial matrix pH to cytosolic pH changes is accounted by P_i_/H^+^ symport and K^+^/H^+^ exchanger. However, the data regarding the effect of agonist-induced [Ca^2+^]_c_ elevations on mitochondrial pH are conflicting, as both acidification and alkalization of matrix were reported [129,130,131]. In their excellent study, Poburko et al. provided several lines of evidence that PMCA was the source of H^+^ during [Ca^2+^]_c_-dependent mitochondrial acidification. First, the acidification matched the kinetic property expected for Ca^2+^/H^+^ antiporter coupling Ca^2+^ extrusion to the proportional load of H^+^; second, the acidification was attributed to Ca^2+^ fluxes at the plasma membrane but not to Ca^2+^ release from intracellular stores; third, alkaline extracellular pH or La^3+^, both known to inhibit PMCA activity, prevented cytosolic acidification. The transmission of acid generated by PMCA to mitochondria may constitute a mechanism, protecting cells from calcium excess, as low pH_mito_ is known to inhibit Ca^2+^-dependent mPTP opening and reduce ROS generation [132,133]. 

Our concurrent measurements of pH_cyto_ and pH_mito_ showed, however, alkalization of mitochondria matrix in steady-state conditions in response to PMCA2 or PMCA3 knockdown (7.78 ± 0.01, 7.62 ± 0.01, respectively, vs. 7.53 ± 0.02 in control). Because pH_cyto_ was also higher in PMCA-deficient cells, but still lower than pH_mito_, which is consistent with chemiosmotic coupling hypothesis, pH gradient across the inner mitochondrial membrane (ΔpH= pH_mito_ − pH_cyto_) was increased in these cells. Based on the PMCA/pH relationship, we anticipate that knockdown of neuro-specific PMCA isoforms, which are considered as fast reacting, dramatically limits the number of H^+^ entering cytosol leading to pH_mito_ increase. In these circumstances, the rise in pH_mito_ could result from compensation of Ca^2+^ charge by moving H^+^ down the ETC complexes. It is therefore attractive to propose that increased [Ca^2+^]_c_ observed in PMCA2-deficient cells, and hence higher [Ca^2+^]_m_, is a main trigger for increased proton motive force to drive higher rate of ATP synthesis. To support this, application of FCCP which collapses the inner mitochondrial membrane potential, evoked significant rise in [Ca^2+^]_c_ in PMCA-deficient lines suggesting higher ΔΨm-dependent mitochondrial Ca^2+^ loading, especially when PMCA2 was knockdown. The phenomenon of higher magnitude of [Ca^2+^]_c_ rises in response to FCCP was also demonstrated by Behringer and colleagues in old microvascular endothelium, suggesting greater capacity of mitochondria for Ca^2+^ during aging [134]. However, respiration-dependent Ca^2+^ entry is expected to lower ΔΨm, as clearly observed in isolated mitochondria exposed to supraphysiological levels of extramitochondrial Ca^2+^ [135]. Under physiological conditions, Ca^2+^ uptake would not always alter ΔΨm, which was demonstrated in isolated cardiomyocytes or it can provoke short-lasting ΔΨm decreases that would not inhibit ATP synthesis [136]. Transient and small fluctuations in ΔΨm in response to cytosolic Ca^2+^ peaks were indeed observed in other systems [137], suggesting plausible cell-specific effect of Ca^2+^ on ΔΨm-driven ATP synthesis. In line with that, Jouaville and colleagues [138] in their key paper suggested that mitochondrial ATP production can be differentially modulated in response to different Ca^2+^ signals generated be various external stimuli. In HeLa cells, that are glycolytic similar to PC12 cells, they demonstrated large increases in [Ca^2+^]_c_ and [ATP]_m_ and concomitant stable rise in ΔΨ_m_ upon histamine stimulation. This suggested that increased [ATP]_m_ reflected long-lasting elevation of the mitochondrial energy state. Similarly, increase in both ΔΨ_m_ and ΔpH components of the proton motive force was seen after stimulation with vasopressin [139], which is known to produce cytosolic [Ca^2+^]c transients that are relayed to mitochondria. The authors suggested that respiratory chain activity may be regulated by a long-lasting mitochondrial signal generated by [Ca^2+^]_m_ transients and thus, partially by [Ca^2+^]_c_ levels, which is coherent with our data from PMCA2-depleted line. These findings also fit the mitochondrial memory mechanism proposed by Jouaville et al. [138] stating that long-term priming of mitochondrial activity persists longer than the rise in [Ca^2+^]_m_. This allows mitochondria to meet the energy needs without the risk of organelle Ca^2+^ overload. Although, the mechanistic explanation of such “memory” is unknown, it plausibly involves the changes in ΔΨ_m,_ as observed by us and/or Ca^2+^-dependent activation of F_0_F_1_ATP synthase. Indeed, several in vitro and in vivo studies demonstrated that the rate of ATP synthesis can be controlled directly at the level of F_0_F_1_ATP synthase, independently from the respiratory rate and ΔΨm [140,141]. F_0_F_1_ATP synthase can bind Ca^2+^ directly [142] but its activity can be also regulated by phosphorylation of the γ-subunit, which in turn is driven by mitochondrial Ca^2+^ [143]. Moreover, a recent study has shown that S100A1 protein binds F_0_F_1_ATP synthase in Ca^2+^-dependent fashion, increasing cellular capacity for ATP synthesis [144], thus providing another way for ΔΨm-independent regulation of energy generation. 

Contrary, none of the PMCA-deficient lines showed a correlation between [Ca^2+^]_c_ response to FCCP and mitochondrial membrane hyperpolarization. Instead, FCCP produced massive loss of ΔΨm and slight hyperpolarization of plasma membrane suggesting that the plasma membrane potential is not created by proton pump and the protonophore equilibrates pH across the plasma membrane by carrying H^+^ from cytoplasm to the extracellular milieu. It has been demonstrated that lack of protons in mitochondrial matrix under the excess of electron donors strongly promotes ROS generation [133]. Therefore, taking into account higher pH_mito_ in PMCA-deficient cells, increased accumulation of Ca^2+^ by mitochondria consisted with the observation of over-activation of ETC complexes, our results point to a specific coupling between PMCA2 generated calcium fluxes and metabolic activity and may mirror specific cellular alterations observed in early aging.

## 5. PMCA and Ca^2+^-Regulated Proteins—CaM, GAP43, CaN in Ageing

The main function of PMCA is to maintain intracellular Ca^2+^ homeostasis, but it is now well documented that efficiency of this control is modulated by several independent regulatory mechanisms [145]. Among them, activation of PMCA by calmodulin (CaM) is the most prominent one, and PMCA is the only calcium pump directly regulated by CaM [146]. Both “fast” PMCA isoforms with a high basal activity exhibit 5 to 10-fold higher affinity for CaM compared to PMCA1 and PMCA4, but are weakly stimulated by CaM [20,147,148]. In rats, CaM is encoded by three non-allelic genes—*Calm1*, *Calm2*, and *Calm3*—all ultimately producing the identical CaM protein. The total CaM amount varies among tissues but the highest concentration (over 30 μM) can be detected in the brain [149]. Because CaM can regulate nearly 300 proteins, there is a high competition rate for CaM binding which is dictated by the affinity of its particular protein partners [150]. Such competition is thought to regulate frequently contradictory processes that are concurrently activated in response to [Ca^2+^]_c_ rises. The prevalence in activation of certain CaM downstream signaling pathways may therefore sensitize neurons to aging-related physiological and pathological stimuli. Like PMCA, CaM shows functional defects in the aged brain, mainly due to intensive oxidation of multiple methionines [2]. Oxidatively modified CaM can still bind PMCA, but cannot stimulate pump activity. Moreover, it also prevents PMCA activation by unoxidized CaM. Although CaM half-life is approximately 18 ± 2 hours, these global structural CaM alterations provide a possible mechanism for the loss of proper calcium regulation during aging [32,151]. 

Early studies on PC12 cells suggested the inversed relationship between CaM level and neurite elongation in response to a differentiation signal [152]. In our studies, we observed decreased CaM immunoreactivity in both PMCA-deficient lines and proposed that the underlying mechanism involved differential regulation of CaM gene expression [153]. Interestingly, the expression of *Calm1* and *Calm2* was downregulated in both modified lines but PMCA2 depletion additionally reduced the expression of *Calm3*. Since PMCA2 and PMCA3 have high basal activity, but are less sensitive to CaM stimulation, limited CaM availability could result in decreased activation of other PMCA isoforms. As a consequence, impaired total PMCA extruding activity can lead to permanent rise in [Ca^2+^]_i_, more significantly in the PC12­_2 line. 

An additional physiological mechanism that can affect CaM function is a formation of complex with growth-associated protein 43 (GAP43, neuromodulin), which is also a marker of differentiating neurons [154,155]. A unique function of this multifunctional presynaptic protein is CaM binding, thereby it can control CaM availability in the cell. Especially high levels of GAP43 were reported at specific sites such as neuronal growth cones, where GAP43 may play a role of abundant CaM reservoir [156]. Since GAP43 half-life is about 2–3 days, this period may be sufficient for reprogramming of CaM-dependent signaling pathways in the cell. Critical for this process is the relationship between binding of CaM and protein kinase C (PKC)-mediated phosphorylation of GAP43. Dephosphorylated GAP43 binds CaM at low Ca^2+^ concentration, while phosphorylation by PKC at Ser 41 liberates CaM due to significant reduction in GAP43 affinity. Dephosphorylation by e.g., calcineurin (CaN), reverses this process and decreases available free CaM [156]. This mechanism directly controls the functional CaM level in the cell, and indirectly all Ca^2+^/CaM-dependent processes. 

In neurons, GAP-43 is distributed throughout all cellular compartments with the highest density found in axon terminals and is implicated in synapse formation and neurite branching [154,157,158]. It has been proposed that the level of GAP-43 might determine the progress of neurogenesis, particularly in the hippocampus and associate cortex in the adult human brain [159]. Physiologically, GAP43 is linked with synaptic plasticity, regulation of neurotransmitter release, learning, and mnemonic function [156]. Behavioral studies demonstrated that reduced GAP-43 level led to some deficits, while increased GAP-43 level enhanced behavioral performance [160]. A study on rats indicated the contribution of this protein to the general age-dependent decline in brain plasticity [161]. During aging, less GAP43 mRNA was detected in the hippocampus, and the phosphorylation level decreased by nearly 50% [162]. Another study on rats reported a lowered GAP43 immunoreactivity in the dentate gyrus, cingulate cortex, and olfactory bulb [163,164,165]. In humans, GAP43 expression predominates during the first decade of life and remains nearly unchanged during further life [166]. However, in Alzheimer’s disease (AD) reduction of neuronal GAP-43 mRNA and GAP-43 protein has been linked to memory dysfunction [167,168]. 

Our studies showed increased GAP43 mRNA and protein level in both PMCA-deficient lines [169]. Interestingly, we detected a higher amount of dephosphorylated GAP43 protein, while its phosphorylation level (P-GAP43) was significantly decreased. Higher GAP43/P-GAP43 ratio indicates enhanced binding of CaM by dephosphorylated form of GAP43. Indeed, in PMCA-deficient lines, a much stronger fluorescent signal from GAP43 and calmodulin-specific antibodies was detected suggesting a more intensive formation of the GAP43/CaM complex whichwas further confirmed by co-immunoprecipitation. Both lowered CaM presence detected in these lines and stronger formation of CaM/GAP43 complex may additionally decrease CaM regulatory potency. 

Further studies using cyclosporine A, an inhibitor of serine-threonine phosphatase—calcineurin (CaN), showed that formation of CaM/GAP43 is controlled by CaN activity [169]. CaN is the only reported phosphatase completely dependent on Ca^2+^/CaM, and it constitutes slightly more than 1% of all brain proteins [170]. In neurons, CaN can be detected in cytoplasm, but also in nearly all subcellular structures i.e., endoplasmic reticulum, Golgi apparatus, nucleus, synaptic vesicles, microsomes, outer mitochondrial membrane, and plasma membrane [30]. Distribution of CaN in the cell appears to be an important factor regulating its local activity. CaN controls numerous and diverse cellular processes mainly via activation of NFAT transcription factor (nuclear factor of activated T cells). Higher activity of calcineurin was observed in aging, as well as in rat and mice models of AD [171,172], and likewise other Ca^2+^/CaM -regulated enzymes, it was linked to an increase in [Ca^2+^]_i_. Some data suggested the relationship between CaN/NFAT-dependent regulation of PMCA and brain aging, along with the onset of neurodegenerative diseases [40,42,173].

To elucidate whether altered composition of PMCA isoforms could affect the expression and activity of CaN in PC12 cells, molecular and biochemical analyses were performed [169]. Using the real-time PCR technique, we detected up-regulation of CaN mRNA by approximately 60% and 85% in PMCA2 or PMCA3-deficient lines, respectively. These changes were also seen at the protein level and gave rise to higher CaN activity. Albeit, there was no direct interaction between GAP43 and PMCA, diminished calcium pumping activity considerably affected CaN and CaM functions. 

One of the physiologically important CaN actions is an orchestration of a sequence of signaling events leading to dephosphorylation of conserved phosphoserine residues in the transcription factor NFAT, its translocation to the nucleus, and subsequent transcriptional activation of NFAT-regulated target genes [170]. However, besides the role of CaN in transcriptional signaling, increasing the number of studies reported the interaction of CaN with other substrates, targeting proteins and regulators of CaN activity. Among others, CaN was shown to interact directly with PMCA2 and PMCA4 isoforms, which resulted in the inhibition of CaN-regulated processes, including activation of NFAT [174,175,176,177]. On the other hand, the expression of PMCA4b in neurons was shown to be controlled by CaN [178]. In our model of differentiated PC12 cells, we detected strong interaction between PMCA2 and CaN. However, high-resolution confocal imaging supported by precipitation of PMCA/CaN immunocomplexes, demonstrated lower degree of colocalization and significantly decreased CaN binding to PMCA in PC12_2 cells. The same set of experiments showed lack of detectable changes in the ability of PMCA2/CaN complex formation between control and PC12_3 cells. Interestingly, in both PMCA-deficient lines, lower PMCA4/CaN immunoreactivity was present, suggesting that PMCA depletion may increase a pool of potentially active CaN. In this context, fast acting PMCA2 and PMCA3 isoforms may be seen as regulators of local CaN availability and function. Control of local CaN activity is beneficial for cell survival in the condition of calcium overload and together with reported protective role of PMCA4 [145,146,148] may be of paramount importance for effective anti-apoptotic protection in PMCA3-deficient line. 

Taken together, changes in PC12 cell lines initiated by moderate, but prolonged increase in [Ca^2+^]_c_ due to specific PMCAs depletion produced distinct cell responses, which clearly depended on PMCA isoforms composition and isoform-specific regulatory mechanisms. When induced changes are adaptive, the cells can effectively counteract Ca^2+^-dependent destructive processes and retain the ability to survive. However, these protective actions in PMCA2 down-regulated cells were found to be much less efficient. One can assume that not only changes in global [Ca^2+^]_I_ concentration, but also spatially and temporally limited modifications in transmission of calcium signal played a decisive role in the cell response, and the detrimental effects over time were not sufficiently counterbalanced by these adaptive mechanisms.

## 6. PMCA-Deficient PC12 Cells as a Tool for Modeling Functional Adaptive Strategies in Neurons during Aging

As described above, aberrant PMCA-mediated Ca^2+^ clearance was associated with perturbation in mechanisms responsible for maintenance of calcium homeostasis. A summary of the detected changes in the expression of analyzed components in PC12_ and PC12_3 are presented in Table 1. As profound changes in CaM expression and availability demonstrated in PMCA-deficient cells could significantly alter CaN activation and CaN-downstream processes, we turned our attention toward a potential cross talk between PMCA, CaM, and CaN in shaping of the Ca^2+^ signal. One of the first basic question we asked was the role of the functional interaction between these proteins in the differential regulation of CaM transcripts and whether this may create a feedback loop for PMCA function. 

Looking for a mechanism(s) that could underlie the diminished expression of CaM genes, a microarray screening of fundamental transcription factors was performed [153]. Among hundreds of analyzed genes, only the expression of *Nfatc2*, whose activity is associated with Ca^2+^, increased in both PC12-deficient lines, which led us to the hypothesis that NFATc2 may function as a transcriptional regulator of calmodulin genes. The NFAT family is composed of five proteins, of which NFATc1, NFATc2, NFATc3, and NFATc4 are activated by Ca^2+^ and their action is controlled by calcineurin [179,180]. In steady-state conditions, NFAT resides in the cytosol in the phosphorylated state, but increased [Ca^2+^]_c_ activates CaN, which dephosphorylates NFAT causing its translocation to the nucleus, where it becomes transcriptionally active. A return of [Ca^2+^]_c_ to the resting level initiates re-phosphorylation and rapid relocation of NFAT back to the cytosol. Our results showed that moderately increased basal [Ca^2+^]_c_ in PMCA-deficient lines sufficiently induced its nuclear translocation and DNA binding activity. Moreover, we found that a sequence for NFATc2 binding is located in the promoter region of *Calm2* and *Calm3* genes, but not in *Calm1*. Chromatin immunoprecipitation assay revealed higher promoter occupancy by NFATc2 in PMCA-modified cell lines resulting in diminished transcription of both CaM genes. In addition, overexpression of the constitutively active form of CaN increased NFATc2 nuclear accumulation and its promoter activity, which potentiated *Calm2* and *Calm3* repression. This strongly indicated the NFATc2 repressive role toward CaM gene expression. Further experiments with NFATc2 silencing, using selective siRNA, showed a partial rescue of the expression of *Calm2* in both lines and *Calm3* in PC12_2 cells, and confirmed that the activation of the CaN/NFAT pathway may repress CaM genes, but to various extent in each of the PMCA-deficient lines. 

The differences in PMCA isoform ratio could affect the regulation of the downstream events including CaN/NFAT-dependent regulation of *Calm 2* and *Calm3* genes. It was reported that CaN interacts with PMCA2 and PMCA4 which resulted in inhibition of its phosphatase activity [175]. In line with it, lowered PMCA2 amount could be partially responsible for diverse cell response. The second important player and limiting factor was the amount of CaM available for binding. This could profoundly interfere with CaN/NFAT activation in both PC12-deficient lines, further suggesting the existence of the feedback mechanism by which CaM could affect its own expression. This specific regulation seems to be a direct consequence of selective PMCA isoform silencing, because no similar effect was observed in the control PC12 cells. Finally, lowered CaM level may have potential consequences on Ca^2+^ extrusion by PMCA, as was reported in senescence neurons [30,32,181]. In addition, since aging was shown to be associated with excessive Ca^2+^ influx through L-type VGCC, which is inactivated by Ca^2+^/CaM complex and directly modulated by CaN [64,182,183], reduced CaM level may thus potentiate calcium influx and inhibit CaN activity as well. In the context of neuronal aging, these results shed new light on molecular basis of neurodegenerative diseases and demonstrated several lines of cellular protection from the negative effects of Ca^2+^ overload. In addition to the membrane components, the maintenance of calcium homeostasis is coupled with the multifunctional endoplasmic reticulum, which contains several Ca^2+^ sensitive transporters, including sarco/endoplasmic Ca^2+^-ATPase (SERCA), inositol 1,4,5-triphosphate receptors (IP_3_Rs), and ryanodine receptors (RyRs). Whereas SERCA decreases [Ca^2+^]_c_ by the uptake into endoplasmic reticulum, IP_3_R and RyR act as channels releasing calcium from the ER following physiological stimulation. In PMCA-reduced cells, an increased level of SERCA2 and SERCA3 coexisted with higher Ca^2+^ accumulation in the ER, although the relationship between PMCA and SERCA expression has not been elucidated. More effective Ca^2+^ transport to the ER may decrease [Ca^2+^]_c_ to its safe level, but also more Ca^2+^ could be released by activation of IP_3_R and RyR [184]. 

IP_3_ receptors are intracellular ubiquitously expressed Ca^2+^ channels that exist in three main isoforms: IP_3_R-1, IP_3_R-2, and IP_3_R-3. In the central nervous system, the presence of all isoforms, with the predominance of IP_3_R-1, was detected, although their subcellular compartmentalization varied in different brain regions [185,186,187]. In the rat brain, IP_3_R-1 was found in high amounts in Purkinje neurons in cerebellum and was localized to dendrites, dendritic spines, cell bodies, axons, and axonal terminals [188,189]. In the hippocampus, IP_3_R-1 is mostly expressed in the CA1 region, with substantially less expression in CA3 and only moderate levels in the granule cells of the dentate gyrus [185]. A particular role of IP_3_Rs in the hippocampus is related to learning and memory abilities, and changes in the IP_3_R isoform composition during aging may have an impact on increased deficits in these processes [190]. In other type of neurons, a high level of IP_3_R-1 was found in cell bodies and proximal dendrites. IP_3_R-2 was mostly detected in glia, whereas IP_3_R-3 was predominantly expressed in neuronal terminals in limbic and basal forebrain regions [191]. The expression of particular receptors during aging is differentially regulated, also in a brain region-specific manner [192,193]. Moreover, IP_3_Rs are dynamically regulated by the formation of homo- or heterotetrameric complexes, thus their relative expression together with other components will determine the final cell response [194,195]. It has been proposed that the level of IP_3_ receptors declines progressively during aging. However, due to the oxidative modifications that are found to increase IP_3_R function in the brain, IP_3_ downstream signaling may not be compromised which is thought to represent a compensation for an altered redox state [186]. Thus, diverse processing of each IP_3_R isoform may activate various cell signaling pathways that can initiate the adaptive response or lead to cell death [187,193]. Since IP_3_R-mediated Ca^2+^ release from the ER and mitochondrial Ca^2+^ homeostasis are physiologically coupled, their improper cooperation may significantly affect cell viability [196].

Release of Ca^2+^ from the ER by IP_3_ receptors requires binding of both, IP_3_—arising from stimulation of phospholipase C (PLC), and Ca^2+^. All isoforms exhibit specific characteristics: IP_3_R-1 possesses low Ca^2+^ affinity and moderate affinity for IP_3_, IP_3_R-2 represents the isoform with the highest affinity for both Ca^2+^ and IP_3_, and IP_3_R-3 is the most sensitive for modulation by Ca^2+^, but displays the lowest IP_3_ affinity [194,195,197]. Interestingly, cytosolic Ca^2+^ regulates IP_3_Rs in a biphasic way: low Ca^2+^ level stimulates the receptors, but the concentration above 300 nM inhibits their transport activity [197]. Our analysis showed that PC12 cells, like the majority of available cell lines, express all three main IP_3_Rs with IP_3_R-3 being the most prominent subtype [198], although IP_3_R-1 and IP_3_R-2 were also detected [193]. In PMCA-deficient cells IP_3_R-1 and IP_3_R-2 expression decreased, whereas IP_3_R-3 was higher (by 40%) than in the control, matching the changes at the protein level. Because this relationship occurred at the transcriptional level, our data could suggest the existence of Ca^2+^-dependent negative feedback loop for IP_3_R-1 and IP_3_R-2 expression. Western blot analysis using an antibody recognizing all IP_3_R isoforms revealed higher total protein level only in the PC12_3 line, pointing out a switch between IP_3_Rs ratio in response to PMCA depletion. Such changes, together with higher presence of PMCA4 and highly active PMCA2, may represent an additional cellular defence mechanism aimed in response to PMCA3 depletion to overcome potentially harmful consequences of calcium excitotoxicity. PMCA4 seems to be central for this defence as it can bind plasma membrane phosphatidylinositol 4,5-bisphosphate (PIP_2_), protecting it from hydrolysis by PLC, potentially decreasing IP_3_ production and, subsequently, Ca^2+^ release from the ER [199]. Some of the adaptive mechanisms revealed in our studies could be relevant to the physiological events occurring during aging, but long-lasting Ca^2+^ dyshomeostasis may promote a variety of potentially detrimental consequences including proteolytic processes triggering apoptosis and/or necrosis.

The wide spectrum of these processes can be propagated by pro-inflammatory chemokines [200] such as CCL5, which induces phospholipase C-mediated liberation of Ca^2+^ from the ER by IP_3_-gated channels. CCL5 can mobilize cytosolic Ca^2+^ after binding to three receptors—CCR1, CCR3, and CCR5, which are cell surface-associated, immune-regulatory G protein-coupled receptors (GPCRs) [201,202]. CCR5 activity has been implicated in normal cell development, but up-regulation of CCR5 was observed in a number of neurological disorders and models of CNS injury [203,204]. Over-activation of CCR5 with subsequent rise in cytosolic Ca^2+^ is known to affect chemotaxis, secretion, and gene expression and could lead to inflammatory and degenerative processes in the CNS [205]. Moreover, higher CCR5 expression in T cells during aging was demonstrated in humans and rodents at both mRNA and protein levels [206,207,208]. It should be noted that higher CCR5 activity may intensify not only CCL5-dependent events, but also the signaling induced by other known CCR5 ligands such as CCL3 and CCL4, of which expression is stimulated in the aging brain [209,210].

All three receptors were detected in our experimental model cells, but only CCR5 was localized in the plasma membrane which is required for CCL5 action. Interestingly, in each of the PMCA down-regulated PC12 lines, CCR5 expression increased by nearly 50%. Functional assays in Ca^2+^-free medium showed that binding of CCL5 to its membrane receptor mobilized Ca^2+^ from the ER more intensively in PMCA-deficient lines but the highest amplitude of [Ca^2+^]_c_ rises was detected in PC12­­_2 cells. In addition, there were significant differences between lines with respect to the rate of Ca^2+^ clearance and the duration of the prolonged calcium signal upon chemokine stimulation. The return of Ca^2+^ to its new steady-state level was 3 times longer in PC12_2 and about 2 times longer in PC12_3 than in control cells. The differences in kinetic parameters could reflect actual rate of Ca^2+^ release from the ER and its extrusion to extracellular milieu. It is worth to emphasize that PMCA3-deficient cells still possess very active PMCA2, but also higher protein level of PMCA4, thus could operate more effectively to control [Ca^2+^]_i_. Despite that, the efficiency of this adaptation is still lower than in control PC12 cells with a full set of PMCA isoforms. 

**Figure 2:**
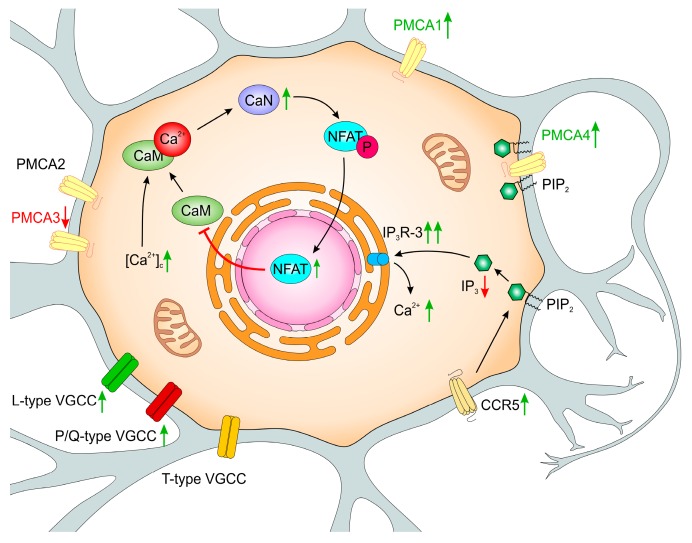
Functional changes in PMCA3-deficient PC12 cells. PMCA3 silencing increased [Ca^2+^]_i_, although PMCA1 and PMCA4 were up-regulated as compensatory effect, but it did not change the total amount of PMCA protein. Increased expression of L- and P/Q types of voltage-gated Ca^2+^ channels (VGCCs) could also raise [Ca^2+^]_i_. The observed up-regulation of CaN affected transcriptional activity of NFATc2, which repressed CaM genes and finally decreased available CaM. Since CaN was also shown to interact directly with PMCA2 and PMCA4 isoforms, the noticed lower PMCA4/CaN complex formation (not shown) could further increase a pool of potentially active CaN. The amount of CCR5 increased in PC12_3 cells, which is linked to IP_3_-mediated Ca^2+^ release from endoplasmic reticulum (ER). The altered ratio of IP_3_Rs expression with the enlarged total quantity, could further increase [Ca^2+^]_i_. However, PMCA4 was shown to bind plasma membrane PIP_2_, potentially decreasing IP_3_ production and, subsequently, Ca^2+^ release from the ER. All these changes, together with higher presence of PMCA4 and highly active PMCA2, may overcome potentially harmful consequences of calcium excitotoxicity. Green up-arrows reflect the increase and red down-arrows reflect the decrease of the amount or activity. Other details are in the text.

**Figure 3:**
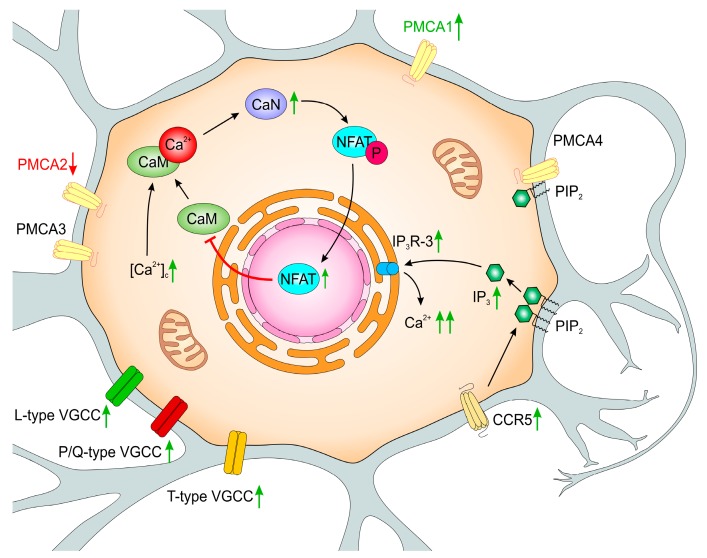
Functional changes in PMCA2-deficient PC12 cells. Down-regulation of PMCA2 increased [Ca^2+^]_i_ due to lowered calcium extrusion potency, despite compensatory up-regulation of PMCA1 and unchanged total PMCA protein level. Elevated expression of VGCC types, particularly T-type that exhibits slow deactivation rate, might contribute to the Ca^2+^ influx. Up-regulated CaN affected transcriptional activity of NFATc2, which repressed CaM genes and lowered CaM amount. Limited CaM availability could weaken its stimulatory effect on PMCA. The amount of CCR5 increased in PC12_2, also with subsequent IP_3_-mediated Ca^2+^ release from ER. Altered ratio of IP_3_Rs expression with higher in the presence of IP_3_R-3, the most sensitive for modulation by Ca^2+^, but with the lowest IP_3_ affinity, could further increase [Ca^2+^]_i_. PMCA2 depletion induced some adaptive changes to counteract Ca^2+^-dependent destructive processes, but because of the enlarged percentage of apoptotic cells, these protective actions appeared to be much less efficient. Green up-arrows reflect the increase and red down-arrows reflect the decrease of the amount or activity. Other details are in the text.

Structural and functional changes in PMCA-deficient PC12 cell lines were initiated by moderately increased cytosolic Ca^2+^, but downstream long-time effects seemed to be specifically linked to the function of particular isoforms. Some of the induced adaptive mechanisms were sufficient for protection of cell viability as observed in PMCA3-deficient cells (Figure 2) in comparison to less effective compensatory changes aimed in response to PMCA2 silencing (Figure 3). This underlines the unique role(s) of PMCA2 and PMCA3 in neuronal cells and indicates that long-lasting calcium dyshomeostasis due to diminished PMCA activity might be central to the aging process and variety of neuropathologies. 

## 7. Conclusions

Aging, as a physiological process, involves the perpetual adaptation to varying internal and external conditions by generation of the complex protective mechanisms. This new adaptive state enables the cells to continuously make short-term adjustments for optimal functioning, but because of the multifactorial nature of aging, the efficiency of these adaptations depends on the intensity of senescent signaling [211,212]. Calcium dyshomeostasis is a factor with especially strong destructive potential. Less efficient Ca^2+^ extruding systems and aberrant regulation of downstream effectors could disturb the function of mitochondria and endoplasmic reticulum leading to cell death, as was widely documented in aging cells [122,213]. 

Studies performed on our PC12 cell model of senescent neurons revealed an apparent contribution of PMCA2 and PMCA3 isoforms to the aging process. A controlled deficiency of PMCA2 or PMCA3 and resulting permanent increase in intracellular Ca^2+^, allowed to track unique functions that each of these isoforms may potentially perform in aging. The most important findings demonstrate that the altered composition of PMCA isoforms is linked to:

- intracellular pH regulation and changes in the mitochondrial bioenergetic processes,
- adaptive functional changes in the VGCC and in Ca^2+^ transport systems to the ER,
- regulation of CaM availability by Ca^2+^/CaN-dependent complex formation with GAP43,
- reduced CaM level and repressive regulation of *Calm2* and *Calm3* genes by NFATc2,
- altered expression of CCL5-sensitive receptors—CCR1, CCR3, and CCR5,
- altered expression of IP_3_ receptors.

Comparison between PC12_2 and PC12_3 lines clearly indicates that down regulation of PMCA2, apart from several adaptive or compensatory processes, triggers more potentially damaging events that are also observed during neuronal aging, including higher sensitivity to inflammation. A declining potency to maintain cellular homeostasis as a potential contributor to age-dependent neuronal senescence markedly increases the risk for neurodegenerative processes. Although differentiated PC12 cells can only partially mimic the physiological processes in the functional neuron, the changes observed in our model might represent the age-related mechanisms common in functionally different cell types. 

## Figures and Tables

**Table 1:** Summary of differences between PMCA2 and PMCA3 down regulation in PC12 cells.

Protein or *mRNA*	PC12­_2	PC12­_3
PMCA1	increased	increased
PMCA2	decreased	unchanged
PMCA3	unchanged	decreased
PMCA4	unchanged	increased
Total PMCA	unchanged	unchanged
SERCA2	increased	increased
SERCA3	increased	increased
CaM	decreased	decreased
CaN	increased	increased
*L-type VGCC*	increased	increased
*P/Q-type VGCC*	increased	increased
*T-type VGCC*	increased	unchanged
CCR1	increased	unchanged
CCR3	unchanged	increased
CCR5	increased	increased
IP­_3_R1	decreased	decreased
IP­_3_R2	decreased	decreased
IP­_3_R3	increased	increased
Total IP­_3_R	unchanged	increased

## References

[1] Chandran R., Kumar M., Kesavan L., Jacob R.S., Gunasekaran S., Lakshmi S., Sadasivan C., Omkumar R.V. (2019). Cellular calcium signaling in the aging brain. J. Chem. Neuroanat..

[2] Squier T.C., Bigelow D.J. (2000). Protein oxidation and age-dependent alterations in calcium homeostasis. Front. Biosci..

[3] Toescu E.C., Vreugdenhil M. (2010). Calcium and normal brain ageing. Cell Calcium.

[4] Labunskyy V.M., Gladyshev V.N. (2013). Role of reactive oxygen species-mediated signaling in aging. Antioxid. Redox. Signal..

[5] Kumar A., Yegla B., Foster T.C. (2018). Redox Signaling in Neurotransmission and Cognition During Aging. Antioxid. Redox. Signal..

[6] Kumar A. (2020). Calcium Signaling During Brain Aging and Its Influence on the Hippocampal Synaptic Plasticity. Adv. Exp. Med. Biol..

[7] Nikoletopoulou V., Tavernarakis N. (2012). Calcium homeostasis in aging neurons. Front. Genet..

[8] Kumar A., Bodhinathan K., Foster T.C. (2009). Susceptibility to Calcium Dysregulation during Brain Aging. Front. Aging. Neurosci..

[9] Kawamoto E.M., Vivar C., Camandola S. (2012). Physiology and pathology of calcium signaling in the brain. Front. Pharmacol..

[10] Lopreiato R., Giacomello M., Carafoli E. (2014). The plasma membrane calcium pump: New ways to look at an old enzyme. J. Biol. Chem..

[11] Stafford N., Wilson C., Oceandy D., Neyses L., Cartwright E.J. (2017). The Plasma Membrane Calcium ATPases and Their Role as Major New Players in Human Disease. Physiol. Rev..

[12] Gunter T.E., Buntinas L., Sparagna G., Eliseev R., Gunter K. (2000). Mitochondrial calcium transport: Mechanisms and functions. Cell Calcium.

[13] Frazier H.N., Maimaiti S., Anderson K.L., Brewer L.D., Gant J.C., Porter N.M., Thibault O. (2017). Calcium’s role as nuanced modulator of cellular physiology in the brain. Biochem. Biophys. Res. Commun..

[14] Muller M., Ahumada-Castro U., Sanhueza M., Gonzalez-Billault C., Court F.A., Cardenas C. (2018). Mitochondria and Calcium Regulation as Basis of Neurodegeneration Associated With Aging. Front. Neurosci..

[15] Akbar M., Essa M.M., Daradkeh G., Abdelmegeed M.A., Choi Y., Mahmood L., Song B.J. (2016). Mitochondrial dysfunction and cell death in neurodegenerative diseases through nitroxidative stress. Brain Res..

[16] Wang C.H., Wu S.B., Wu Y.T., Wei Y.H. (2013). Oxidative stress response elicited by mitochondrial dysfunction: Implication in the pathophysiology of aging. Exp. Biol. Med..

[17] Barja G. (2013). Updating the mitochondrial free radical theory of aging: An integrated view, key aspects, and confounding concepts. Antioxid. Redox. Signal..

[18] Harman D. (1965). The Free Radical Theory of Aging: Effect of Age on Serum Copper Levels. J. Gerontol..

[19] Strehler E.E., Zacharias D.A. (2001). Role of alternative splicing in generating isoform diversity among plasma membrane calcium pumps. Physiol. Rev..

[20] Brini M., Carafoli E. (2009). Calcium pumps in health and disease. Physiol. Rev..

[21] Burette A., Rockwood J.M., Strehler E.E., Weinberg R.J. (2003). Isoform-specific distribution of the plasma membrane Ca^2+^ ATPase in the rat brain. J. Comp. Neurol..

[22] Di Leva F., Domi T., Fedrizzi L., Lim D., Carafoli E. (2008). The plasma membrane Ca^2+^ ATPase of animal cells: Structure, function and regulation. Arch. Biochem. Biophys..

[23] Brini M., Carafoli E. (2011). The plasma membrane Ca^2+^ ATPase and the plasma membrane sodium calcium exchanger cooperate in the regulation of cell calcium. Cold Spring Harb. Perspect. Biol..

[24] Stauffer T.P., Hilfiker H., Carafoli E., Strehler E.E. (1993). Quantitative analysis of alternative splicing options of human plasma membrane calcium pump genes. J. Biol. Chem..

[25] Stauffer T.P., Guerini D., Carafoli E. (1995). Tissue distribution of the four gene products of the plasma membrane Ca^2+^ pump. A study using specific antibodies. J. Biol. Chem..

[26] Zacharias D.A., Kappen C. (1999). Developmental expression of the four plasma membrane calcium ATPase (PMCA) genes in the mouse. Biochim. Biophys. Acta.

[27] Brini M., Cali T., Ottolini D., Carafoli E. (2014). Neuronal calcium signaling: Function and dysfunction. Cell. Mol. Life. Sci..

[28] Sepulveda M.R., Hidalgo-Sanchez M., Marcos D., Mata A.M. (2007). Developmental distribution of plasma membrane Ca^2+^-ATPase isoforms in chick cerebellum. Dev. Dyn..

[29] Kip S.N., Gray N.W., Burette A., Canbay A., Weinberg R.J., Strehler E.E. (2006). Changes in the expression of plasma membrane calcium extrusion systems during the maturation of hippocampal neurons. Hippocampus.

[30] Jiang L., Bechtel M.D., Galeva N.A., Williams T.D., Michaelis E.K., Michaelis M.L. (2012). Decreases in plasma membrane Ca^2+^-ATPase in brain synaptic membrane rafts from aged rats. J. Neurochem..

[31] Zaidi A., Gao J., Squier T.C., Michaelis M.L. (1998). Age-related decrease in brain synaptic membrane Ca^2+^-ATPase in F344/BNF1 rats. Neurobiol. Aging..

[32] Michaelis M.L., Bigelow D.J., Schoneich C., Williams T.D., Ramonda L., Yin D., Huhmer A.F., Yao Y., Gao J., Squier T.C. (1996). Decreased plasma membrane calcium transport activity in aging brain. Life Sci..

[33] Ferrington D.A., Chen X., Krainev A.G., Michaelis E.K., Bigelow D.J. (1997). Protein half-lives of calmodulin and the plasma membrane Ca-ATPase in rat brain. Biochem. Biophys. Res. Commun..

[34] Zaidi A. (2010). Plasma membrane Ca-ATPases: Targets of oxidative stress in brain aging and neurodegeneration. World J. Biol. Chem..

[35] Zaidi A., Michaelis M.L. (1999). Effects of reactive oxygen species on brain synaptic plasma membrane Ca^2+^-ATPase. Free Radic. Biol. Med..

[36] Kip S.N., Strehler E.E. (2007). Rapid downregulation of NCX and PMCA in hippocampal neurons following H_2_O_2_ oxidative stress. Ann. N. Y. Acad. Sci..

[37] Giacomello M., De Mario A., Lopreiato R., Primerano S., Campeol M., Brini M., Carafoli E. (2011). Mutations in PMCA2 and hereditary deafness: A molecular analysis of the pump defect. Cell Calcium.

[38] Cali T., Lopreiato R., Shimony J., Vineyard M., Frizzarin M., Zanni G., Zanotti G., Brini M., Shinawi M., Carafoli E. (2015). A Novel Mutation in Isoform 3 of the Plasma Membrane Ca^2+^ Pump Impairs Cellular Ca^2+^ Homeostasis in a Patient with Cerebellar Ataxia and Laminin Subunit 1α Mutations. J. Biol. Chem..

[39] Zanni G., Cali T., Kalscheuer V.M., Ottolini D., Barresi S., Lebrun N., Montecchi-Palazzi L., Hu H., Chelly J., Bertini E. (2012). Mutation of plasma membrane Ca^2+^ ATPase isoform 3 in a family with X-linked congenital cerebellar ataxia impairs Ca^2+^ homeostasis. Proc. Natl. Acad. Sci. USA.

[40] Berrocal M., Marcos D., Sepulveda M.R., Perez M., Avila J., Mata A.M. (2009). Altered Ca^2+^ dependence of synaptosomal plasma membrane Ca^2+^-ATPase in human brain affected by Alzheimer’s disease. FASEB J..

[41] Berrocal M., Sepulveda M.R., Vazquez-Hernandez M., Mata A.M. (2012). Calmodulin antagonizes amyloid-β peptides-mediated inhibition of brain plasma membrane Ca^2+^-ATPase. Biochim. Biophys. Acta.

[42] Berrocal M., Corbacho I., Vazquez-Hernandez M., Avila J., Sepulveda M.R., Mata A.M. (2015). Inhibition of PMCA activity by tau as a function of aging and Alzheimer’s neuropathology. Biochim. Biophys. Acta.

[43] Berrocal M., Corbacho I., Sepulveda M.R., Gutierrez-Merino C., Mata A.M. (2017). Phospholipids and calmodulin modulate the inhibition of PMCA activity by tau. Biochim. Biophys. Acta Mol. Cell. Res..

[44] Greene L.A., Tischler A.S. (1976). Establishment of a noradrenergic clonal line of rat adrenal pheochromocytoma cells which respond to nerve growth factor. Proc. Natl. Acad. Sci. USA.

[45] Keller D., Grover A.K. (2000). Nerve growth factor treatment alters Ca^2+^ pump levels in PC12 cells. Neuroreport.

[46] Lambeng N., Michel P.P., Agid Y., Ruberg M. (2001). The relationship between differentiation and survival in PC12 cells treated with cyclic adenosine monophosphate in the presence of epidermal growth factor or nerve growth factor. Neurosci. Lett..

[47] Hammes A., Oberdorf S., Strehler E.E., Stauffer T., Carafoli E., Vetter H., Neyses L. (1994). Differentiation-specific isoform mRNA expression of the calmodulin-dependent plasma membrane Ca^2+^-ATPase. FASEB J..

[48] Garcia M.L., Usachev Y.M., Thayer S.A., Strehler E.E., Windebank A.J. (2001). Plasma membrane calcium ATPase plays a role in reducing Ca^2+^-mediated cytotoxicity in PC12 cells. J. Neurosci. Res..

[49] Boczek T., Lisek M., Kowalski A., Pikula S., Niewiarowska J., Wiktorska M., Zylinska L. (2012). Downregulation of PMCA2 or PMCA3 reorganizes Ca^2+^ handling systems in differentiating PC12 cells. Cell Calcium.

[50] Luo L., O’Leary D.D. (2005). Axon retraction and degeneration in development and disease. Annu. Rev. Neurosci..

[51] Baranov S.V., Baranova O.V., Yablonska S., Suofu Y., Vazquez A.L., Kozai T.D.Y., Cui X.T., Ferrando L.M., Larkin T.M., Tyurina Y.Y. (2019). Mitochondria modulate programmed neuritic retraction. Proc. Natl. Acad. Sci. USA.

[52] Fernandes D., Zaidi A., Bean J., Hui D., Michaelis M.L. (2007). RNA_i_—Induced silencing of the plasma membrane Ca^2+^-ATPase 2 in neuronal cells: Effects on Ca^2+^ homeostasis and cell viability. J. Neurochem..

[53] Kurnellas M.P., Li H., Jain M.R., Giraud S.N., Nicot A.B., Ratnayake A., Heary R.F., Elkabes S. (2010). Reduced expression of plasma membrane calcium ATPase 2 and collapsin response mediator protein 1 promotes death of spinal cord neurons. Cell. Death Differ..

[54] Empson R.M., Turner P.R., Nagaraja R.Y., Beesley P.W., Knopfel T. (2010). Reduced expression of the Ca^2+^ transporter protein PMCA2 slows Ca^2+^ dynamics in mouse cerebellar Purkinje neurones and alters the precision of motor coordination. J. Physiol..

[55] Raza M., Deshpande L.S., Blair R.E., Carter D.S., Sombati S., DeLorenzo R.J. (2007). Aging is associated with elevated intracellular calcium levels and altered calcium homeostatic mechanisms in hippocampal neurons. Neurosci. Lett..

[56] Mata A.M., Sepulveda M.R. (2010). Plasma membrane Ca-ATPases in the nervous system during development and ageing. World J. Biol. Chem..

[57] Strehler E.E., Thayer S.A. (2018). Evidence for a role of plasma membrane calcium pumps in neurodegenerative disease: Recent developments. Neurosci. Lett..

[58] Carafoli E., Brini M. (2000). Calcium pumps: Structural basis for and mechanism of calcium transmembrane transport. Curr. Opin. Chem. Biol..

[59] Mata A.M., Sepulveda M.R. (2005). Calcium pumps in the central nervous system. Brain Res. Rev..

[60] MacLennan D.H., Rice W.J., Green N.M. (1997). The mechanism of Ca^2+^ transport by sarco(endo)plasmic reticulum Ca^2+^-ATPases. J. Biol. Chem..

[61] Janigro D., Maccaferri G., Meldolesi J. (1989). Calcium channels in undifferentiated PC12 rat pheochromocytoma cells. FEBS Lett.

[62] Liu H., Felix R., Gurnett C.A., De Waard M., Witcher D.R., Campbell K.P. (1996). Expression and subunit interaction of voltage-dependent Ca^2+^ channels in PC12 cells. J. Neurosci..

[63] Strickland M., Yacoubi-Loueslati B., Bouhaouala-Zahar B., Pender S.L.F., Larbi A. (2019). Relationships between Ion Channels, Mitochondrial Functions and Inflammation in Human Aging. Front. Physiol..

[64] Thibault O., Landfield P.W. (1996). Increase in single L-type calcium channels in hippocampal neurons during aging. Science.

[65] Campbell L.W., Hao S.Y., Thibault O., Blalock E.M., Landfield P.W. (1996). Aging changes in voltage-gated calcium currents in hippocampal CA1 neurons. J. Neurosci..

[66] Brewer L.D., Thibault O., Staton J., Thibault V., Rogers J.T., Garcia-Ramos G., Kraner S., Landfield P.W., Porter N.M. (2007). Increased vulnerability of hippocampal neurons with age in culture: Temporal association with increases in NMDA receptor current, NR2A subunit expression and recruitment of L-type calcium channels. Brain Res..

[67] Thibault O., Gant J.C., Landfield P.W. (2007). Expansion of the calcium hypothesis of brain aging and Alzheimer’s disease: Minding the store. Aging Cell.

[68] Navakkode S., Liu C., Soong T.W. (2018). Altered function of neuronal L-type calcium channels in ageing and neuroinflammation: Implications in age-related synaptic dysfunction and cognitive decline. Ageing Res. Rev..

[69] Thibault O., Hadley R., Landfield P.W. (2001). Elevated postsynaptic [Ca^2+^]_i_ and L-type calcium channel activity in aged hippocampal neurons: Relationship to impaired synaptic plasticity. J. Neurosci..

[70] Zamponi G.W., Striessnig J., Koschak A., Dolphin A.C. (2015). The Physiology, Pathology, and Pharmacology of Voltage-Gated Calcium Channels and Their Future Therapeutic Potential. Pharmacol. Rev..

[71] Rice R.A., Berchtold N.C., Cotman C.W., Green K.N. (2014). Age-related downregulation of the CaV_3.1_ T-type calcium channel as a mediator of amyloid beta production. Neurobiol. Aging.

[72] Del Toro R., Levitsky K.L., Lopez-Barneo J., Chiara M.D. (2003). Induction of T-type calcium channel gene expression by chronic hypoxia. J. Biol. Chem..

[73] Baker D.J., Peleg S. (2017). Biphasic Modeling of Mitochondrial Metabolism Dysregulation during Aging. Trends Biochem. Sci..

[74] Bratic A., Larsson N.G. (2013). The role of mitochondria in aging. J. Clin. Investig..

[75] Ziegler D.V., Wiley C.D., Velarde M.C. (2015). Mitochondrial effectors of cellular senescence: Beyond the free radical theory of aging. Aging Cell.

[76] Lopes G.S., Ferreira A.T., Oshiro M.E., Vladimirova I., Jurkiewicz N.H., Jurkiewicz A., Smaili S.S. (2006). Aging-related changes of intracellular Ca^2+^ stores and contractile response of intestinal smooth muscle. Exp. Gerontol..

[77] Buchholz J.N., Behringer E.J., Pottorf W.J., Pearce W.J., Vanterpool C.K. (2007). Age-dependent changes in Ca^2+^ homeostasis in peripheral neurones: Implications for changes in function. Aging Cell.

[78] Friel D.D. (2000). Mitochondria as regulators of stimulus-evoked calcium signals in neurons. Cell Calcium.

[79] Verkhratsky A., Toescu E.C. (1998). Calcium and neuronal ageing. Trends Neurosci..

[80] Martinez-Serrano A., Blanco P., Satrustegui J. (1992). Calcium binding to the cytosol and calcium extrusion mechanisms in intact synaptosomes and their alterations with aging. J. Biol. Chem..

[81] Kirischuk S., Verkhratsky A. (1996). Calcium homeostasis in aged neurones. Life Sci..

[82] Denton R.M., Randle P.J., Bridges B.J., Cooper R.H., Kerbey A.L., Pask H.T., Severson D.L., Stansbie D., Whitehouse S. (1975). Regulation of mammalian pyruvate dehydrogenase. Mol. Cell. Biochem..

[83] McKenzie M., Lim S.C., Duchen M.R. (2017). Simultaneous Measurement of Mitochondrial Calcium and Mitochondrial Membrane Potential in Live Cells by Fluorescent Microscopy. J. Vis. Exp..

[84] Madreiter-Sokolowski C.T., Sokolowski A.A., Graier W.F. (2017). *Dosis Facit Sanitatem*—Concentration-Dependent Effects of Resveratrol on Mitochondria. Nutrients.

[85] Gauba E., Guo L., Du H. (2017). Cyclophilin D Promotes Brain Mitochondrial F1FO ATP Synthase Dysfunction in Aging Mice. J. Alzheimers Dis..

[86] Panel M., Ghaleh B., Morin D. (2018). Mitochondria and aging: A role for the mitochondrial transition pore?. Aging Cell.

[87] Scorrano L., Petronilli V., Bernardi P. (1997). On the voltage dependence of the mitochondrial permeability transition pore. A critical appraisal. J. Biol. Chem..

[88] Boczek T., Kozaczuk A., Taha J., Ferenc B., Zylinska L. (2010). Adaptation of microsomal glutathione transferase 1 in PC12 cells with modified PMCA isoforms composition. Indian J. Biochem. Biophys..

[89] Marques-Aleixo I., Rocha-Rodrigues S., Santos-Alves E., Coxito P.M., Passos E., Oliveira P.J., Magalhaes J., Ascensao A. (2012). In vitro salicylate does not further impair aging-induced brain mitochondrial dysfunction. Toxicology.

[90] Krestinina O., Azarashvili T., Baburina Y., Galvita A., Grachev D., Stricker R., Reiser G. (2015). In aging, the vulnerability of rat brain mitochondria is enhanced due to reduced level of 2′,3′-cyclic nucleotide-3′-phosphodiesterase (CNP) and subsequently increased permeability transition in brain mitochondria in old animals. Neurochem. Int..

[91] Pandya J.D., Grondin R., Yonutas H.M., Haghnazar H., Gash D.M., Zhang Z., Sullivan P.G. (2015). Decreased mitochondrial bioenergetics and calcium buffering capacity in the basal ganglia correlates with motor deficits in a nonhuman primate model of aging. Neurobiol. Aging.

[92] Papa S., Martino P.L., Capitanio G., Gaballo A., De Rasmo D., Signorile A., Petruzzella V. (2012). The oxidative phosphorylation system in mammalian mitochondria. Adv. Exp. Med. Biol..

[93] Naghdi S., Waldeck-Weiermair M., Fertschai I., Poteser M., Graier W.F., Malli R. (2010). Mitochondrial Ca^2+^ uptake and not mitochondrial motility is required for STIM1-Orai1-dependent store-operated Ca^2+^ entry. J. Cell Sci..

[94] Waldeck-Weiermair M., Alam M.R., Khan M.J., Deak A.T., Vishnu N., Karsten F., Imamura H., Graier W.F., Malli R. (2012). Spatiotemporal correlations between cytosolic and mitochondrial Ca^2+^ signals using a novel red-shifted mitochondrial targeted cameleon. PLoS ONE.

[95] Raturi A., Simmen T. (2013). Where the endoplasmic reticulum and the mitochondrion tie the knot: The mitochondria-associated membrane (MAM). Biochim. Biophys. Acta.

[96] Kruger W.A., Monteith G.R., Poronnik P. (2010). The plasma membrane Ca^2+^-ATPase: Regulation by PSD-95/Dlg/Zo-1 scaffolds. Int. J. Biochem. Cell Biol..

[97] Garside M.L., Turner P.R., Austen B., Strehler E.E., Beesley P.W., Empson R.M. (2009). Molecular interactions of the plasma membrane calcium ATPase 2 at pre- and post-synaptic sites in rat cerebellum. Neuroscience.

[98] Davies S., Ramsden D.B. (2001). Huntington’s disease. Mol. Pathol..

[99] Lisek M., Ferenc B., Studzian M., Pulaski L., Guo F., Zylinska L., Boczek T. (2017). Glutamate Deregulation in Ketamine-Induced Psychosis-A Potential Role of PSD95, NMDA Receptor and PMCA Interaction. Front. Cell. Neurosci..

[100] Nicholls D.G. (2009). Mitochondrial calcium function and dysfunction in the central nervous system. Biochim. Biophys. Acta.

[101] Pivovarova N.B., Andrews S.B. (2010). Calcium-dependent mitochondrial function and dysfunction in neurons. FEBS J..

[102] Duchen M.R. (2012). Mitochondria, calcium-dependent neuronal death and neurodegenerative disease. Pflugers. Arch..

[103] Qiu J., Tan Y.W., Hagenston A.M., Martel M.A., Kneisel N., Skehel P.A., Wyllie D.J., Bading H., Hardingham G.E. (2013). Mitochondrial calcium uniporter Mcu controls excitotoxicity and is transcriptionally repressed by neuroprotective nuclear calcium signals. Nat. Commun..

[104] Zoratti M., Szabo I. (1995). The mitochondrial permeability transition. Biochim. Biophys. Acta.

[105] Wang R., Reddy P.H. (2017). Role of Glutamate and NMDA Receptors in Alzheimer’s Disease. J. Alzheimers Dis..

[106] Li V., Wang Y.T. (2016). Molecular mechanisms of NMDA receptor-mediated excitotoxicity: Implications for neuroprotective therapeutics for stroke. Neural Regen. Res..

[107] Zhou Q., Sheng M. (2013). NMDA receptors in nervous system diseases. Neuropharmacology.

[108] Pagliarini D.J., Calvo S.E., Chang B., Sheth S.A., Vafai S.B., Ong S.E., Walford G.A., Sugiana C., Boneh A., Chen W.K. (2008). A mitochondrial protein compendium elucidates complex I disease biology. Cell.

[109] Sancak Y., Markhard A.L., Kitami T., Kovacs-Bogdan E., Kamer K.J., Udeshi N.D., Carr S.A., Chaudhuri D., Clapham D.E., Li A.A. (2013). EMRE is an essential component of the mitochondrial calcium uniporter complex. Science.

[110] Kamer K.J., Mootha V.K. (2015). The molecular era of the mitochondrial calcium uniporter. Nat. Rev. Mol. Cell. Biol..

[111] Kamer K.J., Jiang W., Kaushik V.K., Mootha V.K., Grabarek Z. (2019). Crystal structure of MICU2 and comparison with MICU1 reveal insights into the uniporter gating mechanism. Proc. Natl. Acad. Sci. USA.

[112] Mallilankaraman K., Doonan P., Cardenas C., Chandramoorthy H.C., Muller M., Miller R., Hoffman N.E., Gandhirajan R.K., Molgo J., Birnbaum M.J. (2012). MICU1 is an essential gatekeeper for MCU-mediated mitochondrial Ca^2+^ uptake that regulates cell survival. Cell.

[113] Csordas G., Golenar T., Seifert E.L., Kamer K.J., Sancak Y., Perocchi F., Moffat C., Weaver D., de la Fuente Perez S., Bogorad R. (2013). MICU1 controls both the threshold and cooperative activation of the mitochondrial Ca^2+^ uniporter. Cell Metab..

[114] Patron M., Checchetto V., Raffaello A., Teardo E., Vecellio Reane D., Mantoan M., Granatiero V., Szabo I., De Stefani D., Rizzuto R. (2014). MICU1 and MICU2 finely tune the mitochondrial Ca^2+^ uniporter by exerting opposite effects on MCU activity. Mol. Cell..

[115] Liu J.C., Liu J., Holmstrom K.M., Menazza S., Parks R.J., Fergusson M.M., Yu Z.X., Springer D.A., Halsey C., Liu C. (2016). MICU1 Serves as a Molecular Gatekeeper to Prevent In Vivo Mitochondrial Calcium Overload. Cell Rep..

[116] Rasmussen T.P., Wu Y., Joiner M.L., Koval O.M., Wilson N.R., Luczak E.D., Wang Q., Chen B., Gao Z., Zhu Z. (2015). Inhibition of MCU forces extramitochondrial adaptations governing physiological and pathological stress responses in heart. Proc. Natl. Acad. Sci. USA.

[117] Konig T., Troder S.E., Bakka K., Korwitz A., Richter-Dennerlein R., Lampe P.A., Patron M., Muhlmeister M., Guerrero-Castillo S., Brandt U. (2016). The m-AAA Protease Associated with Neurodegeneration Limits MCU Activity in Mitochondria. Mol. Cell..

[118] Plovanich M., Bogorad R.L., Sancak Y., Kamer K.J., Strittmatter L., Li A.A., Girgis H.S., Kuchimanchi S., De Groot J., Speciner L. (2013). MICU2, a paralog of MICU1, resides within the mitochondrial uniporter complex to regulate calcium handling. PLoS ONE.

[119] Markus N.M., Hasel P., Qiu J., Bell K.F., Heron S., Kind P.C., Dando O., Simpson T.I., Hardingham G.E. (2016). Expression of mRNA Encoding Mcu and Other Mitochondrial Calcium Regulatory Genes Depends on Cell Type, Neuronal Subtype, and Ca^2+^ Signaling. PLoS ONE.

[120] Patron M., Granatiero V., Espino J., Rizzuto R., De Stefani D. (2019). MICU3 is a tissue-specific enhancer of mitochondrial calcium uptake. Cell Death Differ..

[121] Calvo-Rodriguez M., Garcia-Durillo M., Villalobos C., Nunez L. (2016). In vitro aging promotes endoplasmic reticulum (ER)-mitochondria Ca^2+^ cross talk and loss of store-operated Ca^2+^ entry (SOCE) in rat hippocampal neurons. Biochim. Biophys. Acta.

[122] Madreiter-Sokolowski C.T., Waldeck-Weiermair M., Bourguignon M.P., Villeneuve N., Gottschalk B., Klec C., Stryeck S., Radulovic S., Parichatikanond W., Frank S. (2019). Enhanced inter-compartmental Ca^2+^ flux modulates mitochondrial metabolism and apoptotic threshold during aging. Redox Biol..

[123] Madreiter-Sokolowski C.T., Sokolowski A.A., Waldeck-Weiermair M., Malli R., Graier W.F. (2018). Targeting Mitochondria to Counteract Age-Related Cellular Dysfunction. Genes.

[124] Manczak M., Jung Y., Park B.S., Partovi D., Reddy P.H. (2005). Time-course of mitochondrial gene expressions in mice brains: Implications for mitochondrial dysfunction, oxidative damage, and cytochrome c in aging. J. Neurochem..

[125] Itoh K., Weis S., Mehraein P., Muller-Hocker J. (1996). Cytochrome c oxidase defects of the human substantia nigra in normal aging. Neurobiol. Aging.

[126] Bertoni-Freddari C., Fattoretti P., Giorgetti B., Solazzi M., Balietti M., Casoli T., Di Stefano G. (2004). Cytochrome oxidase activity in hippocampal synaptic mitochondria during aging: A quantitative cytochemical investigation. Ann. N. Y. Acad. Sci..

[127] Poburko D., Santo-Domingo J., Demaurex N. (2011). Dynamic regulation of the mitochondrial proton gradient during cytosolic calcium elevations. J. Biol. Chem..

[128] Balut C., vandeVen M., Despa S., Lambrichts I., Ameloot M., Steels P., Smets I. (2008). Measurement of cytosolic and mitochondrial pH in living cells during reversible metabolic inhibition. Kidney Int..

[129] Bolshakov A.P., Mikhailova M.M., Szabadkai G., Pinelis V.G., Brustovetsky N., Rizzuto R., Khodorov B.I. (2008). Measurements of mitochondrial pH in cultured cortical neurons clarify contribution of mitochondrial pore to the mechanism of glutamate-induced delayed Ca^2+^ deregulation. Cell Calcium.

[130] Malli R., Frieden M., Osibow K., Zoratti C., Mayer M., Demaurex N., Graier W.F. (2003). Sustained Ca^2+^ transfer across mitochondria is Essential for mitochondrial Ca^2+^ buffering, sore-operated Ca^2+^ entry, and Ca^2+^ store refilling. J. Biol. Chem..

[131] Abad M.F., Di Benedetto G., Magalhaes P.J., Filippin L., Pozzan T. (2004). Mitochondrial pH monitored by a new engineered green fluorescent protein mutant. J. Biol. Chem..

[132] Petronilli V., Cola C., Bernardi P. (1993). Modulation of the mitochondrial cyclosporin A-sensitive permeability transition pore. II. The minimal requirements for pore induction underscore a key role for transmembrane electrical potential, matrix pH, and matrix Ca^2+^. J. Biol. Chem..

[133] Selivanov V.A., Zeak J.A., Roca J., Cascante M., Trucco M., Votyakova T.V. (2008). The role of external and matrix pH in mitochondrial reactive oxygen species generation. J. Biol. Chem..

[134] Behringer E.J., Segal S.S. (2017). Impact of Aging on Calcium Signaling and Membrane Potential in Endothelium of Resistance Arteries: A Role for Mitochondria. J. Gerontol. A Biol. Sci. Med. Sci..

[135] Gunter T.E., Pfeiffer D.R. (1990). Mechanisms by which mitochondria transport calcium. Am. J. Physiol..

[136] Tarasov A.I., Griffiths E.J., Rutter G.A. (2012). Regulation of ATP production by mitochondrial Ca^2+^. Cell Calcium.

[137] Luciani D.S., Misler S., Polonsky K.S. (2006). Ca^2+^ controls slow NAD(P)H oscillations in glucose-stimulated mouse pancreatic islets. J. Physiol..

[138] Jouaville L.S., Pinton P., Bastianutto C., Rutter G.A., Rizzuto R. (1999). Regulation of mitochondrial ATP synthesis by calcium: Evidence for a long-term metabolic priming. Proc. Natl. Acad. Sci. USA.

[139] Robb-Gaspers L.D., Burnett P., Rutter G.A., Denton R.M., Rizzuto R., Thomas A.P. (1998). Integrating cytosolic calcium signals into mitochondrial metabolic responses. EMBO J..

[140] Harris D.A., Das A.M. (1991). Control of mitochondrial ATP synthesis in the heart. Biochem. J..

[141] Scholz T.D., Balaban R.S. (1994). Mitochondrial F1-ATPase activity of canine myocardium: Effects of hypoxia and stimulation. Am. J. Physiol..

[142] Hubbard M.J., McHugh N.J. (1996). Mitochondrial ATP synthase F1-β-subunit is a calcium-binding protein. FEBS Lett..

[143] Hopper R.K., Carroll S., Aponte A.M., Johnson D.T., French S., Shen R.F., Witzmann F.A., Harris R.A., Balaban R.S. (2006). Mitochondrial matrix phosphoproteome: Effect of extra mitochondrial calcium. Biochemistry.

[144] Boerries M., Most P., Gledhill J.R., Walker J.E., Katus H.A., Koch W.J., Aebi U., Schoenenberger C.A. (2007). Ca^2+^ -dependent interaction of S100A1 with F1-ATPase leads to an increased ATP content in cardiomyocytes. Mol. Cell. Biol..

[145] Padanyi R., Paszty K., Hegedus L., Varga K., Papp B., Penniston J.T., Enyedi A. (2016). Multifaceted plasma membrane Ca^2+^ pumps: From structure to intracellular Ca^2+^ handling and cancer. Biochim. Biophys. Acta.

[146] Cali T., Brini M., Carafoli E. (2017). Regulation of Cell Calcium and Role of Plasma Membrane Calcium ATPases. Int. Rev. Cell. Mol. Biol..

[147] Elwess N.L., Filoteo A.G., Enyedi A., Penniston J.T. (1997). Plasma membrane Ca^2+^ pump isoforms 2a and 2b are unusually responsive to calmodulin and Ca^2+^. J. Biol. Chem..

[148] Strehler E.E. (2015). Plasma membrane calcium ATPases: From generic Ca^2+^ sump pumps to versatile systems for fine-tuning cellular Ca^2+^. Biochem. Biophys. Res. Commun..

[149] Toutenhoofd S.L., Strehler E.E. (2000). The calmodulin multigene family as a unique case of genetic redundancy: Multiple levels of regulation to provide spatial and temporal control of calmodulin pools?. Cell Calcium.

[150] Shen X., Valencia C.A., Szostak J.W., Dong B., Liu R. (2005). Scanning the human proteome for calmodulin-binding proteins. Proc. Natl. Acad. Sci. USA.

[151] Bigelow D.J., Squier T.C. (2005). Redox modulation of cellular signaling and metabolism through reversible oxidation of methionine sensors in calcium regulatory proteins. Biochim. Biophys. Acta.

[152] Davidkova G., Zhang S.P., Nichols R.A., Weiss B. (1996). Reduced level of calmodulin in PC12 cells induced by stable expression of calmodulin antisense RNA inhibits cell proliferation and induces neurite outgrowth. Neuroscience.

[153] Boczek T., Lisek M., Ferenc B., Zylinska L. (2017). Cross talk among PMCA, calcineurin and NFAT transcription factors in control of calmodulin gene expression in differentiating PC12 cells. Biochim. Biophys. Acta Gene Regul. Mech..

[154] Benowitz L.I., Routtenberg A. (1997). GAP-43: An intrinsic determinant of neuronal development and plasticity. Trends Neurosci..

[155] Denny J.B. (2006). Molecular mechanisms, biological actions, and neuropharmacology of the growth-associated protein GAP-43. Curr. Neuropharmacol..

[156] Holahan M.R. (2017). A Shift from a Pivotal to Supporting Role for the Growth-Associated Protein (GAP-43) in the Coordination of Axonal Structural and Functional Plasticity. Front. Cell. Neurosci..

[157] Donnelly C.J., Park M., Spillane M., Yoo S., Pacheco A., Gomes C., Vuppalanchi D., McDonald M., Kim H.H., Merianda T.T. (2013). Axonally synthesized β-actin and GAP-43 proteins support distinct modes of axonal growth. J. Neurosci..

[158] Yoo S., Kim H.H., Kim P., Donnelly C.J., Kalinski A.L., Vuppalanchi D., Park M., Lee S.J., Merianda T.T., Perrone-Bizzozero N.I. (2013). A HuD-ZBP1 ribonucleoprotein complex localizes GAP-43 mRNA into axons through its 3′ untranslated region AU-rich regulatory element. J. Neurochem..

[159] Neve R.L., Finch E.A., Bird E.D., Benowitz L.I. (1988). Growth-associated protein GAP-43 is expressed selectively in associative regions of the adult human brain. Proc. Natl. Acad. Sci. USA.

[160] Latchney S.E., Masiulis I., Zaccaria K.J., Lagace D.C., Powell C.M., McCasland J.S., Eisch A.J. (2014). Developmental and adult GAP-43 deficiency in mice dynamically alters hippocampal neurogenesis and mossy fiber volume. Dev. Neurosci..

[161] Schmoll H., Ramboiu S., Platt D., Herndon J.G., Kessler C., Popa-Wagner A. (2005). Age influences the expression of GAP-43 in the rat hippocampus following seizure. Gerontology.

[162] Barnes C.A., Mizumori S.J., Lovinger D.M., Sheu F.S., Murakami K., Chan S.Y., Linden D.J., Nelson R.B., Routtenberg A. (1988). Selective decline in protein F1 phosphorylation in hippocampus of senescent rats. Neurobiol. Aging.

[163] Casoli T., Di Stefano G., Gracciotti N., Giovagnetti S., Fattoretti P., Solazzi M., Bertoni-Freddari C. (2001). Cellular distribution of GAP-43 mRNA in hippocampus and cerebellum of adult rat brain by in situ RT-PCR. J. Histochem. Cytochem..

[164] Casoli T., Spagna C., Fattoretti P., Gesuita R., Bertoni-Freddari C. (1996). Neuronal plasticity in aging: A quantitative immunohistochemical study of GAP-43 distribution in discrete regions of the rat brain. Brain Res..

[165] Casoli T., Stefano G.D., Fattoretti P., Solazzi M., Delfino A., Biagini G., Bertoni-Freddari C. (2003). GAP-43 mRNA detection by in situ hybridization, direct and indirect in situ RT-PCR in hippocampal and cerebellar tissue sections of adult rat brain. Micron.

[166] Webster M.J., Elashoff M., Weickert C.S. (2011). Molecular evidence that cortical synaptic growth predominates during the first decade of life in humans. Int. J. Dev. Neurosci..

[167] De la Monte S.M., Ng S.C., Hsu D.W. (1995). Aberrant GAP-43 gene expression in Alzheimer’s disease. Am. J. Pathol..

[168] Rekart J.L., Quinn B., Mesulam M.M., Routtenberg A. (2004). Subfield-specific increase in brain growth protein in postmortem hippocampus of Alzheimer’s patients. Neuroscience.

[169] Boczek T., Ferenc B., Lisek M., Zylinska L. (2015). Regulation of GAP43/calmodulin complex formation via calcineurin-dependent mechanism in differentiated PC12 cells with altered PMCA isoforms composition. Mol. Cell. Biochem..

[170] Kipanyula M.J., Kimaro W.H., Seke Etet P.F. (2016). The Emerging Roles of the Calcineurin-Nuclear Factor of Activated T-Lymphocytes Pathway in Nervous System Functions and Diseases. J. Aging Res..

[171] Abdul H.M., Furman J.L., Sama M.A., Mathis D.M., Norris C.M. (2010). NFATs and Alzheimer’s Disease. Mol. Cell. Pharmacol..

[172] Norris C.M., Kadish I., Blalock E.M., Chen K.C., Thibault V., Porter N.M., Landfield P.W., Kraner S.D. (2005). Calcineurin triggers reactive/inflammatory processes in astrocytes and is upregulated in aging and Alzheimer’s models. J. Neurosci..

[173] Li H., Rao A., Hogan P.G. (2011). Interaction of calcineurin with substrates and targeting proteins. Trends Cell. Biol..

[174] Curry M.C., Luk N.A., Kenny P.A., Roberts-Thomson S.J., Monteith G.R. (2012). Distinct regulation of cytoplasmic calcium signals and cell death pathways by different plasma membrane calcium ATPase isoforms in MDA-MB-231 breast cancer cells. J. Biol. Chem..

[175] Holton M., Yang D., Wang W., Mohamed T.M., Neyses L., Armesilla A.L. (2007). The interaction between endogenous calcineurin and the plasma membrane calcium-dependent ATPase is isoform specific in breast cancer cells. FEBS Lett..

[176] Wu X., Chang B., Blair N.S., Sargent M., York A.J., Robbins J., Shull G.E., Molkentin J.D. (2009). Plasma membrane Ca^2+^-ATPase isoform 4 antagonizes cardiac hypertrophy in association with calcineurin inhibition in rodents. J. Clin. Investig..

[177] Baggott R.R., Mohamed T.M., Oceandy D., Holton M., Blanc M.C., Roux-Soro S.C., Brown S., Brown J.E., Cartwright E.J., Wang W. (2012). Disruption of the interaction between PMCA2 and calcineurin triggers apoptosis and enhances paclitaxel-induced cytotoxicity in breast cancer cells. Carcinogenesis.

[178] Foster T.C., Sharrow K.M., Masse J.R., Norris C.M., Kumar A. (2001). Calcineurin links Ca^2+^ dysregulation with brain aging. J. Neurosci..

[179] Sommerer C., Meuer S., Zeier M., Giese T. (2012). Calcineurin inhibitors and NFAT-regulated gene expression. Clin. Chim. Acta.

[180] Muller M.R., Rao A. (2010). NFAT, immunity and cancer: A transcription factor comes of age. Nat. Rev. Immunol..

[181] Teolato S., Calderini G., Bonetti A.C., Toffano G. (1983). Calmodulin content in different brain areas of aging rats. Neurosci. Lett..

[182] Saimi Y., Kung C. (2002). Calmodulin as an ion channel subunit. Annu. Rev. Physiol..

[183] Norris C.M., Blalock E.M., Chen K.C., Porter N.M., Landfield P.W. (2002). Calcineurin enhances L-type Ca^2+^ channel activity in hippocampal neurons: Increased effect with age in culture. Neuroscience.

[184] Chemaly E.R., Troncone L., Lebeche D. (2018). SERCA control of cell death and survival. Cell Calcium.

[185] Foskett J.K., White C., Cheung K.H., Mak D.O. (2007). Inositol trisphosphate receptor Ca^2+^ release channels. Physiol. Rev..

[186] Decuypere J.P., Monaco G., Missiaen L., De Smedt H., Parys J.B., Bultynck G. (2011). IP_3_ Receptors, Mitochondria, and Ca Signaling: Implications for Aging. J. Aging Res..

[187] Vervloessem T., Yule D.I., Bultynck G., Parys J.B. (2015). The type 2 inositol 1,4,5-trisphosphate receptor, emerging functions for an intriguing Ca^2+^-release channel. Biochim. Biophys. Acta.

[188] Dent M.A., Raisman G., Lai F.A. (1996). Expression of type 1 inositol 1,4,5-trisphosphate receptor during axogenesis and synaptic contact in the central and peripheral nervous system of developing rat. Development.

[189] Mikoshiba K. (2007). IP_3_ receptor/Ca^2+^ channel: From discovery to new signaling concepts. J. Neurochem..

[190] Kumar A., Gibbs J.R., Beilina A., Dillman A., Kumaran R., Trabzuni D., Ryten M., Walker R., Smith C., Traynor B.J. (2013). Age-associated changes in gene expression in human brain and isolated neurons. Neurobiol. Aging.

[191] Sharp A.H., Nucifora F.C., Blondel O., Sheppard C.A., Zhang C., Snyder S.H., Russell J.T., Ryugo D.K., Ross C.A. (1999). Differential cellular expression of isoforms of inositol 1,4,5-triphosphate receptors in neurons and glia in brain. J. Comp. Neurol..

[192] Simonyi A., Xia J., Igbavboa U., Wood W.G., Sun G.Y. (1998). Age differences in the expression of metabotropic glutamate receptor 1 and inositol 1,4,5-trisphosphate receptor in mouse cerebellum. Neurosci. Lett..

[193] Ivanova H., Vervliet T., Missiaen L., Parys J.B., De Smedt H., Bultynck G. (2014). Inositol 1,4,5-trisphosphate receptor-isoform diversity in cell death and survival. Biochim. Biophys. Acta.

[194] Iwai M., Michikawa T., Bosanac I., Ikura M., Mikoshiba K. (2007). Molecular basis of the isoform-specific ligand-binding affinity of inositol 1,4,5-trisphosphate receptors. J. Biol. Chem..

[195] Parys J.B., De Smedt H. (2012). Inositol 1,4,5-trisphosphate and its receptors. Adv. Exp. Med. Biol..

[196] Marchi S., Patergnani S., Missiroli S., Morciano G., Rimessi A., Wieckowski M.R., Giorgi C., Pinton P. (2018). Mitochondrial and endoplasmic reticulum calcium homeostasis and cell death. Cell Calcium.

[197] Berridge M.J. (2016). The Inositol Trisphosphate/Calcium Signaling Pathway in Health and Disease. Physiol. Rev..

[198] Radzik T., Boczek T., Ferenc B., Studzian M., Pulaski L., Zylinska L. (2019). Calcium Dyshomeostasis Alters CCL5 Signaling in Differentiated PC12 Cells. Biomed. Res. Int..

[199] Penniston J.T., Padanyi R., Paszty K., Varga K., Hegedus L., Enyedi A. (2014). Apart from its known function, the plasma membrane Ca^2+^ ATPase can regulate Ca^2+^ signaling by controlling phosphatidylinositol 4,5-bisphosphate levels. J. Cell. Sci..

[200] Sama D.M., Norris C.M. (2013). Calcium dysregulation and neuroinflammation: Discrete and integrated mechanisms for age-related synaptic dysfunction. Ageing Res. Rev..

[201] Marques R.E., Guabiraba R., Russo R.C., Teixeira M.M. (2013). Targeting CCL5 in inflammation. Expert Opin. Ther. Targets.

[202] Pranzatelli M.R. (2018). Advances in Biomarker-Guided Therapy for Pediatric- and Adult-Onset Neuroinflammatory Disorders: Targeting Chemokines/Cytokines. Front. Immunol..

[203] Gamo K., Kiryu-Seo S., Konishi H., Aoki S., Matsushima K., Wada K., Kiyama H. (2008). G-protein-coupled receptor screen reveals a role for chemokine receptor CCR5 in suppressing microglial neurotoxicity. J. Neurosci..

[204] Takeshita Y., Ransohoff R.M. (2012). Inflammatory cell trafficking across the blood-brain barrier: Chemokine regulation and in vitro models. Immunol. Rev..

[205] Balistreri C.R., Caruso C., Grimaldi M.P., Listi F., Vasto S., Orlando V., Campagna A.M., Lio D., Candore G. (2007). CCR5 receptor: Biologic and genetic implications in age-related diseases. Ann. N. Y. Acad. Sci..

[206] Chen J., Mo R., Lescure P.A., Misek D.E., Hanash S., Rochford R., Hobbs M., Yung R.L. (2003). Aging is associated with increased T-cell chemokine expression in C57BL/6 mice. J. Gerontol. A Biol. Sci. Med. Sci..

[207] Naumova E., Ivanova M., Pawelec G. (2011). Immunogenetics of ageing. Int. J. Immunogenet..

[208] Yung R.L., Mo R. (2003). Aging is associated with increased human T cell CC chemokine receptor gene expression. J. Interferon Cytokine Res..

[209] Tripathy D., Thirumangalakudi L., Grammas P. (2007). Expression of macrophage inflammatory protein 1-α is elevated in Alzheimer’s vessels and is regulated by oxidative stress. J. Alzheimers Dis..

[210] Zhu M., Allard J.S., Zhang Y., Perez E., Spangler E.L., Becker K.G., Rapp P.R. (2014). Age-related brain expression and regulation of the chemokine CCL4/MIP-1beta in APP/PS1 double-transgenic mice. J. Neuropathol. Exp. Neurol..

[211] Pomatto L.C.D., Davies K.J.A. (2017). The role of declining adaptive homeostasis in ageing. J. Physiol..

[212] Pomatto L.C.D., Sun P.Y., Davies K.J.A. (2019). To adapt or not to adapt: Consequences of declining Adaptive Homeostasis and Proteostasis with age. Mech. Ageing Dev..

[213] Janikiewicz J., Szymanski J., Malinska D., Patalas-Krawczyk P., Michalska B., Duszynski J., Giorgi C., Bonora M., Dobrzyn A., Wieckowski M.R. (2018). Mitochondria-associated membranes in aging and senescence: Structure, function, and dynamics. Cell. Death Dis..

